# Upregulation of NKG2D ligands impairs hematopoietic stem cell function in Fanconi anemia

**DOI:** 10.1172/JCI142842

**Published:** 2022-08-01

**Authors:** José A. Casado, Antonio Valeri, Rebeca Sanchez-Domínguez, Paula Vela, Andrea López, Susana Navarro, Omaira Alberquilla, Helmut Hanenberg, Roser Pujol, José-Carlos Segovia, Jordi Minguillón, Jordi Surrallés, Cristina Díaz de Heredia, Julián Sevilla, Paula Rio, Juan A. Bueren

**Affiliations:** 1 Division of Innovative Therapies, CIEMAT and Advanced Therapies Unit, IIS-Fundación Jimenez Diaz and Autónoma University, Madrid, Spain.; 2 Biomedical Center for Research on Rare Diseases (CIBERER), Madrid, Spain.; 3 Department of Pediatrics III, University Children’s Hospital Essen, University of Duisburg-Essen, Essen, Germany.; 4 Department of Otorhinolaryngology and Head and Neck Surgery, Heinrich Heine University, Duesseldorf, Germany.; 5 IIB Sant Pau, Hospital de la Santa Creu i Sant Pau, Univ Autònoma de Barcelona, Barcelona Spain.; 6 Servei d’Oncologia i Hematologia Pediàtrica, Hospital Universitari Vall d’Hebron, Barcelona, Spain.; 7 Stem Cell Transplant Unit, Hospital Niño Jesús, Madrid, Spain.

**Keywords:** Immunology, Stem cells, Bone marrow, Cellular immune response, Hematopoietic stem cells

## Abstract

Fanconi anemia (FA) is the most prevalent inherited bone marrow failure (BMF) syndrome. Nevertheless, the pathophysiological mechanisms of BMF in FA have not been fully elucidated. Since FA cells are defective in DNA repair, we hypothesized that FA hematopoietic stem and progenitor cells (HSPCs) might express DNA damage–associated stress molecules such as natural killer group 2 member D ligands (NKG2D-Ls). These ligands could then interact with the activating NKG2D receptor expressed in cytotoxic NK or CD8^+^ T cells, which may result in progressive HSPC depletion. Our results indeed demonstrated upregulated levels of NKG2D-Ls in cultured FA fibroblasts and T cells, and these levels were further exacerbated by mitomycin C or formaldehyde. Notably, a high proportion of BM CD34^+^ HSPCs from patients with FA also expressed increased levels of NKG2D-Ls, which correlated inversely with the percentage of CD34^+^ cells in BM. Remarkably, the reduced clonogenic potential characteristic of FA HSPCs was improved by blocking NKG2D–NKG2D-L interactions. Moreover, the in vivo blockage of these interactions in a BMF FA mouse model ameliorated the anemia in these animals. Our study demonstrates the involvement of NKG2D–NKG2D-L interactions in FA HSPC functionality, suggesting an unexpected role of the immune system in the progressive BMF that is characteristic of FA.

## Graphical abstract

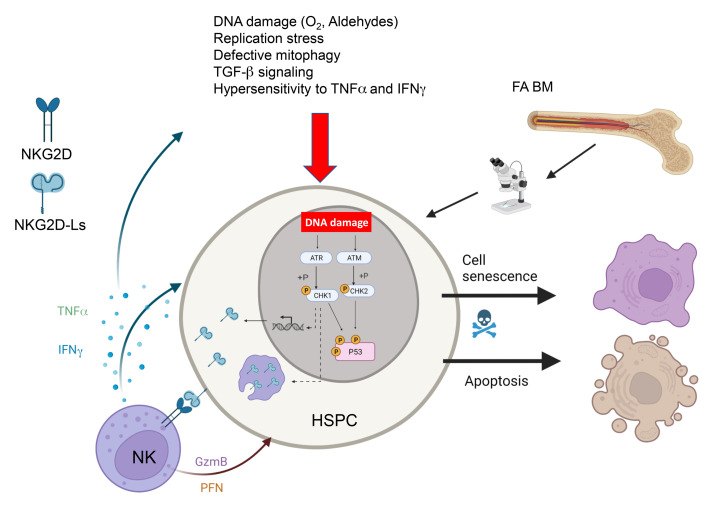

## Introduction

Fanconi anemia (FA) is a rare inherited disorder mainly characterized by congenital abnormalities, bone marrow failure (BMF), and cancer predisposition (1). Currently, allogeneic transplantation of hematopoietic stem and progenitor cells (HSPCs) is the preferential therapeutic option for the rescue of BMF in patients with FA (2). Nevertheless, recent clinical data have shown that HSPC gene therapy may constitute an innovative, low-toxicity therapeutic option for this life-threatening disorder (3).

So far, 23 FA genes have been characterized that encode for proteins participating in the FA/BRCA pathway required for the repair of DNA interstrand crosslinks (ICLs) and for the preservation of genome stability (see reviews in refs. 4, 5). Additionally, it has been proposed that at least some FA proteins participate in other cellular functions, whose alterations can also play a significant role in FA disease (see review in ref. 6).

In an effort to understand the mechanisms involved in FA BMF, previous studies have shown that FA cells are hypersensitive to various cellular stressors, which include oxidative damage (7, 8), endogenous aldehydes (9, 10), and cytotoxic inflammatory cytokines such as TNF-α and IFN-γ, which, furthermore, are present at increased levels in the BM of patients with FA (11, 12). Additionally, elevated TGF-β signaling (13) and insufficient viral clearance in FA cells (14) may account for the BMF characteristic of FA. Despite advances in the understanding of the mechanisms involved in BMF of patients with FA, further studies are required to fully understand the progression of BMF in this disease.

Regarding the involvement of the immune system in BMF diseases, previous studies have indeed implicated cells of the immune system as important factors in acquired BMF syndromes (15, 16). However, there is no reported evidence showing the relevance of specific interactions between immune cells and HSPCs in the progressive BMF that is characteristic of FA. In general, cells of the innate immune system, including NK cells, are generated in BM and migrate to peripheral lymphoid organs, where they mature and undergo education (17). The cytotoxic function of these cells depends on the integration of activation and inhibitory signals from an array of surface receptors (17). NKG2D is an activating homodimeric member of the NKG2 family of C-type lectin–like receptors that enables immune cells to recognize damaged/stressed autologous cells by interaction with cell-surface ligands indicative of cellular distress (18). The human NKG2D receptor is mainly present on NK cells, αβ CD8^+^ T cells, NKT cells, and a subset γδ T cells. When engaged, NKG2D generates one of the strongest activating signals in NK cells and also provides a costimulatory signal in CD8^+^ T cells (19, 20). Ligands of NKG2D (NKG2D-Ls) are 8 membrane proteins MICA, MICB, and ULBP1 to ULBP6, which are generally absent on healthy cells. However, high expression of NKG2D-Ls can typically be found in cancer cells and in somatic cells that either harbor DNA damage or are affected by oxidative stress, viral infection, or other cellular stressors (21–24). Different studies have shown that overexpression of these ligands enables NKG2D^+^ effector cells to efficiently recognize and eliminate NKG2D-L^+^ cells (23, 25). NKG2D–NKG2D-L interactions play an important role in physiological signaling pathways that recognize stressed or damaged cells, thereby enabling specific cytotoxic cells of the innate immune system to remove abnormal cells (26, 27).

Taking into account the profound phenotypic defects of FA cells, including a diminished ability to repair DNA crosslinks, we hypothesized that these cells might express increased levels of NKG2D-Ls. This upregulation of NKG2-Ls on FA HSPCs would promote a deleterious hematopoietic response by immune cells, thus contributing to the progressive BMF observed in most patients. Data presented in this study demonstrates that, compared with healthy cells, cultured fibroblasts and peripheral blood (PB) T cells from patients with FA expressed high levels of NKG2D-Ls, a process that was further exacerbated by DNA cross-linker agents. Moreover, a high proportion of fresh CD34^+^ cells from patients with FA also expressed high levels of NKG2D-Ls, which correlated with low numbers of CD34^+^ cells. These data, together with in vitro as well as in vivo proof-of-principle experiments showing improved hematopoietic effects associated with the blockage of NKG2D–NKG2D-L interactions, demonstrate the relevance of cells of the innate immune system in the impaired growth of FA HSPCs and strongly implicate NKG2D–NKG2D-L interactions in the BMF characteristic of patients with FA.

## Results

### FA fibroblasts and T cells express upregulated levels of NKG2D-Ls in a DNA damage–dependent manner.

**Figure 1:**
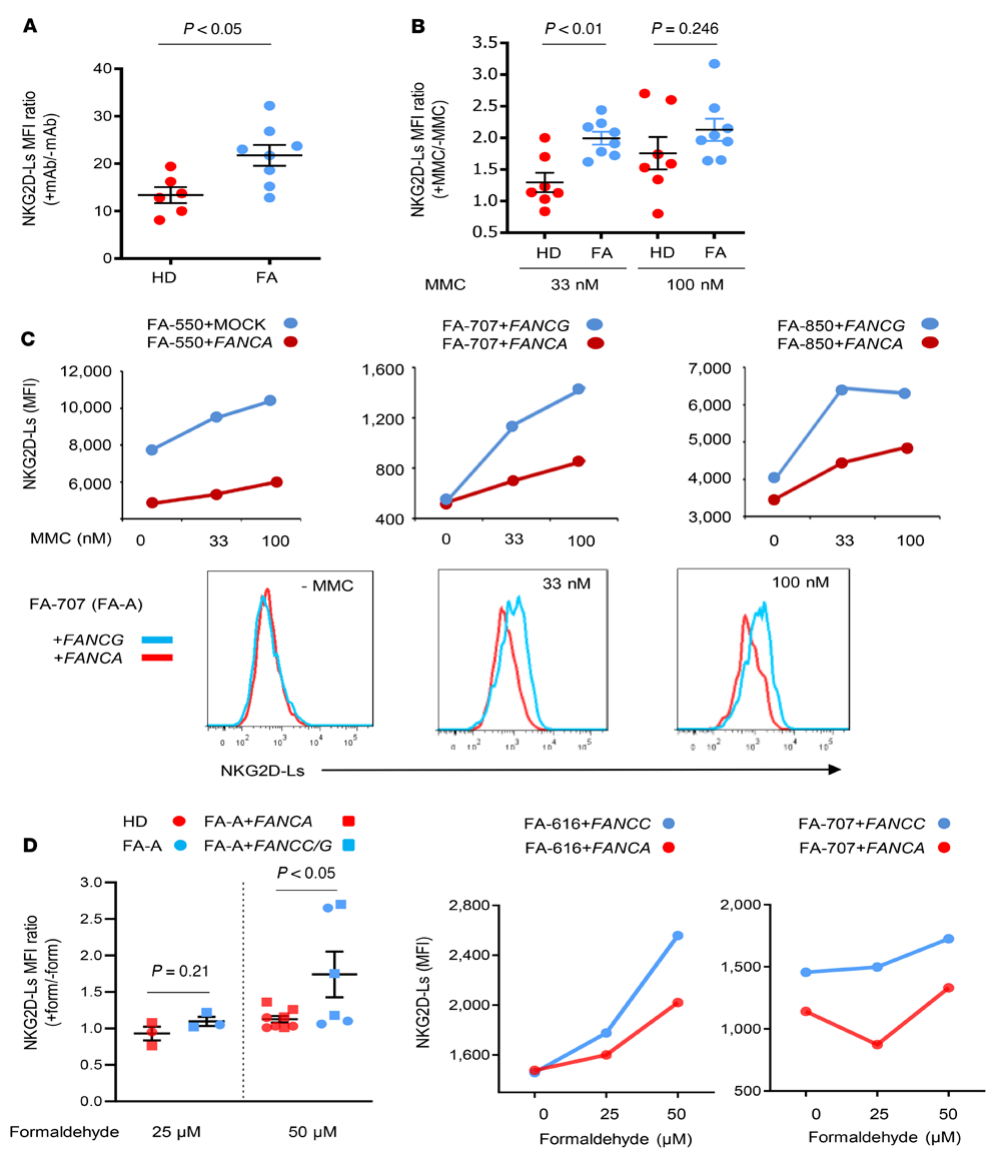
Comparative analysis of NKG2D-L levels in uncorrected and gene-corrected fibroblasts from patients with FA-A and HDs. (**A**) Primary fibroblasts from HDs (*n =* 6) and patients with FA-A (*n =* 8) were cultured at a similar confluence, and NKG2D-L levels were analyzed with a mixture of anti–NKG2D-L antibodies (anti-MICA, -MICB, and –ULBP1-6). Data show the ratio of the MFI of NKG2D-L staining with respect to basal fluorescence. (**B**) Fold increase of NKG2D-L levels in MMC-treated (+MMC) versus untreated (–MMC) fibroblasts from HDs (*n =* 7) and patients with FA-A (*n =* 8). Primary fibroblasts were maintained for 3 days in the absence or presence of MMC (33 nM and 100 nM). NKG2D-L MFI ratios of MMC-treated to nontreated cells are shown. (**C**) Levels of NKG2D-L (MFI values) in uncorrected and gene-corrected fibroblasts from 3 patients with FA-A. The fibroblasts were exposed to 0 nM, 33 nM, or 100 nM MMC. Corrected cells were generated by transduction with a RV carrying the *FANCA* gene (+*FANCA*), whereas uncorrected cells consisted of either untransduced cells (MOCK) or cells transduced with a RV encoding *FANCG* (+*FANCG*). Representative histograms of NKG2D-L levels in fibroblasts from patient FA-707 are also shown (lower panels). (**D**) Fold increase of NKG2D-L levels in formaldehyde-treated fibroblasts versus untreated fibroblasts corresponding to FA-competent cells (HD or corrected FA-A cells; FA-A+*FANCA*) or FA pathway–deficient cells (untransduced; FA-A or *FANCC* or *FANCG*-transduced FA-A cells; FA-A+*FANCC/G*). Data in **A**, **B**, and **D** represent the mean ± SEM. A 2-tailed, unpaired *t* test was used to compare mean values between HD and FA fibroblasts, either corrected or uncorrected.

In order to investigate our hypothesis that FA cells might express elevated levels of NKG2D-Ls, we first evaluated the basal expression of NKG2D-Ls in in vitro–cultured fibroblasts from patients with mutations in *FANCA* (patients of the FA-A complementation group) and from healthy donors (HDs). Since all NKG2D-Ls interact with the same NKG2D receptor (28), cells were stained with a mixture of monoclonal antibodies that recognize the 8 NKG2D-Ls (MICA, MICB, and ULBP1–6), as previously described (29). Flow cytometric analyses of NKG2D-Ls revealed significant increases in the membrane expression of these ligands in FA versus HD fibroblasts (see ratios of the MFI values in Figure 1A and raw MFI data in Supplemental Figure 1A; supplemental material available online with this article; https://doi.org/10.1172/JCI142842DS1). Additionally, we observed that the DNA cross-linker agent mitomycin C (MMC) further increased the differences in NKG2D-L levels between FA and HD fibroblasts (Figure 1B and Supplemental Figure 1A).

Next, we investigated whether the high NKG2D-L levels observed in FA-A cells were a direct consequence of their genetic defect and could therefore be downregulated by the genetic correction of these cells. Studies in complemented and noncomplemented fibroblasts from 3 FA-A patients showed that genetic correction with *FANCA* retroviral vectors (+*FANCA* RVs) reduced NKG2D-L levels when compared with their negative controls (untransduced FA-A cells or cells transduced with a noncorrecting *FANCG* RV (+*FANCG*) (Figure 1C). As expected, the effect of gene complementation on NKG2D-L levels was even more marked when FA cells were exposed to MMC (Figure 1C). Consistent with these studies, enhanced levels of NKG2D-Ls were confirmed in the immunofluorescence analyses conducted in uncorrected FA-A fibroblasts and T cells (Supplemental Figure 1B). We also observed that, as happened with the expression of NKG2D-Ls, the proportion of cells with nuclear γ-H2AX foci, which are indicative of DNA damage, was highest in MMC-treated uncorrected FA fibroblasts (Supplemental Figure 2).

In subsequent studies we evaluated whether NKG2D-L differences between FA and HD fibroblasts were mainly due to the upregulated expression of specific ligands. Among the different NKG2D-Ls, we found that differences between HD and FA fibroblasts were mainly due to the overexpression of MICA, MICB, ULBP-3, and ULBP-4 (Supplemental Figure 3). In contrast to these ligands, no significant ULBP-1 upregulation was observed in FA cells, and in the case of ULBP-2, -5, and, -6, these ligands were already upregulated in HD fibroblasts.

Since aldehydes are physiological metabolites with particular damaging effects in FA cells (10), we investigated whether, as happened with MMC treatment, formaldehyde also induces NKG2D-L overexpression in FA fibroblasts. As shown in Figure 1D, there were significant differences in the induction of NKG2D-Ls in FA fibroblasts treated with 50 μM formaldehyde (see NKG2D-L MFI ratios between formaldehyde-treated and untreated FA fibroblasts). Representative analyses of NKG2D-L levels (MFI values) in untreated and formaldehyde-treated FA fibroblasts — either uncorrected or gene-corrected — are shown in the right panels of Figure 1D. These results confirmed that this endogenous genotoxic agent also resulted in high levels of NKG2D-Ls in FA fibroblasts.

**Figure 2:**
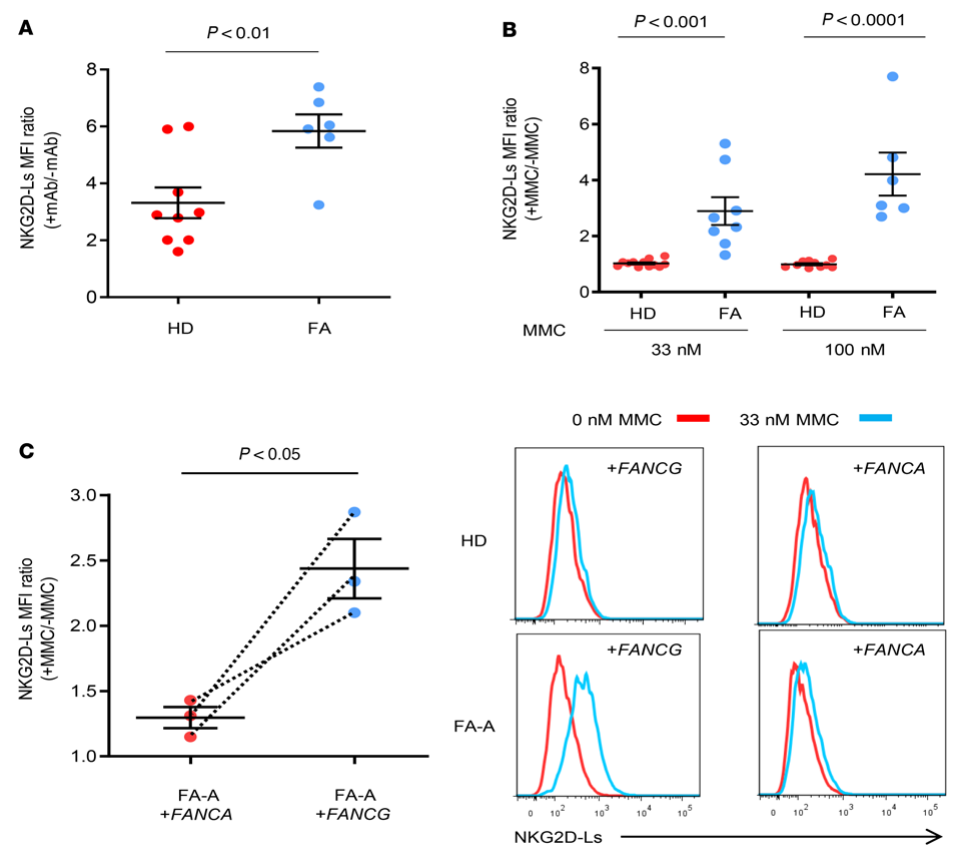
Comparative analysis of NKG2D-L levels in uncorrected and gene-corrected T cells from patients with FA-A and HDs. (**A**) Comparative analysis of NKG2D-Ls in in vitro–cultured T cells from HDs (*n =* 9) and patients with FA-A (*n =* 6). Mononuclear PB cells from HDs and patients with FA-A were stimulated with anti-CD3 and anti-CD28 mAbs for 4 days and analyzed for NKG2D-L expression. The figure shows the ratio of the MFI after staining with NKG2D-L antibodies with respect to the basal fluorescence level. (**B**) Fold increase of NKG2D-L levels in MMC-treated (33 nM or 100 nM) versus untreated T cells from HDs (*n =* 12, 33 nM and *n =* 10, 100 nM) and patients with FA-A (*n =* 8, 33 nM and *n =* 6, 100 nM). NKG2D-L MFI ratios between MMC-treated and untreated cells are shown. (**C**) Analysis of MMC-induced (33 nM) expression of NKG2D-L in uncorrected (+*FANCG*) and corrected (+*FANCA*) T cells from 3 patients with FA-A. Flow cytometric histograms show representative NKG2D-L analyses in HD and FA-A T cells transduced with the *FANCA* or *FANCG* vector. Data in **A**–**C** represent the mean ± SEM. In **A** and **B**, differences between HD and FA cells were assessed by unpaired *t* test. In **C**, differences between corrected and uncorrected samples were evaluated using a paired *t* test.

To analyze the expression levels of NKG2D-Ls in a different somatic cell type, we used PB T cells from HDs and patients with FA. As observed in FA fibroblasts, higher basal levels of NKG2D-Ls were detected in in vitro–cultured T cells from patients with FA when compared with basal levels in HD T cells (Figure 2A and Supplemental Figure 1). Additionally, while no increases in NKG2D-L levels were observed in HD T cells after MMC treatment (MFI ratios of MMC-treated cells to untreated cells were close to 1), this drug significantly increased the levels of NKG2D-Ls in FA T cells (MFI ratios in cells exposed to 33 nM and 100 nM increased 2.9- and 4.2-fold, respectively; Figure 2B and Supplemental Figure 1). To examine the effect of gene complementation on NKG2D-L levels in FA T cells, samples previously transduced with *FANCA* or *FANCG* RVs were maintained for 3 days in the absence or the presence of MMC (33 nM). As shown in Figure 2C, we observed increased levels of NKG2D-Ls when noncomplemented FA-A T cells (transduced with the *FANCG* vector) were exposed to MMC. However, almost no changes in NKG2D-Ls levels were induced by MMC in complemented FA-A cells (Figure 2C). As expected, NKG2D-L levels observed in HD T cells either transduced with *FANCA* or *FANCG* vectors were not modified by exposure to MMC (see representative analyses in Figure 2C).

Taken together, these results demonstrate that, compared with HD cells, primary FA-A fibroblasts and T cells expressed higher levels of NKG2D-Ls. Additionally, our data show that the expression of these markers of cellular damage/stress was further increased after genotoxic damage.

### Upregulation of NKG2D-Ls in fresh BM CD34^+^ cells from patients with FA.

**Figure 3:**
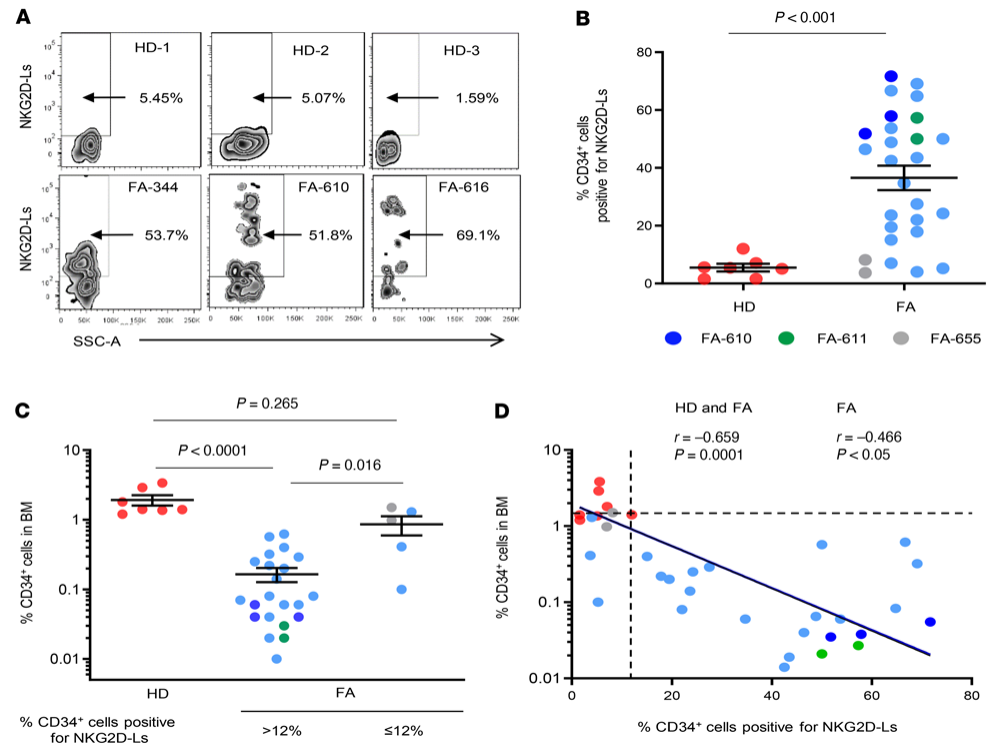
Analysis of NKG2D-L levels in fresh BM CD34^+^ cells from HDs and patients with FA. (**A**) Representative contour plot analyses of NKG2D-L in CD34^+^ cells from HDs (*n =* 3) and patients with FA (*n =* 3). SSC-A, side scatter area. (**B**) Proportion of CD34^+^ cells positive for NKG2D-L in BM from HDs (*n =* 7) and patients with FA (*n =* 23). Light blue data points correspond to FA BM samples from 1 single analysis (*n =* 20). Dark blue (FA-610), green (FA-611), and gray (FA-655) data points correspond to BM samples from the same patient with FA analyzed at different time points (*n =* 27 total FA samples). Additional details on the FA and HD samples are provided in Tables 1 and 2. (**C**) Analysis of the percentage of CD34^+^ cells in BM samples from HDs and patients with FA. FA samples were classified into 2 groups according to NKG2D-L levels, using as a cutoff the maximum percentage of NKG2D-Ls determined in HD samples (12%; see Tables 1 and 2). (HD, *n =* 7; FA >12% NKG2D-L^+^, *n =* 22; FA ≤12% NKG2D-L^+^, *n =* 5). *P* values were determined by Kruskal-Wallis test followed by Dunn’s multiple-comparison test. (**D**) Correlation analysis between the percentage of CD34^+^ cells in BM and the proportion of CD34^+^ cells positive for NKG2D-Ls in samples from HDs and patients with FA (data point colors as in **B**, *n =* 27) and HDs (red data points, *n =* 7). Mean values ± SEM are indicated for each group in **B** and **C**, and significance was calculated by unpaired *t* test. Spearman’s correlation test was applied for assessment of HD and FA samples and also only for FA samples. Dashed lines indicate the lowest percentage of CD34^+^ cells and the highest proportion of NKG2D-L expression in BM CD34^+^ cells from HDs.

In order to investigate whether the expression of NGK2D-Ls was also increased in primary HSPCs from patients with FA, we analyzed fresh BM CD34^+^ cells from 23 nonmosaic FA patients and 7 HDs (Figure 3 and Tables 1 and 2). In some patients (FA-610, FA-611, and FA-655), we performed repeated BM aspirations, which allowed us to include a total of 27 FA BM samples in these studies. In addition to the main characteristics of patients with FA and HDs, Tables 1 and 2 show the actual percentage of CD34^+^ cells in BM, as well as the proportion of CD34^+^ cells positive for NKG2D-L expression.

As expected, the proportion of CD34^+^ cells in BM from patients with FA (0.29% ± 0.08%) was significantly lower than that determined in BM from HDs (1.98% ± 0.37%) (Tables 1 and 2). The expression levels of NKG2D-Ls in HD and FA CD34^+^ cells were also investigated by flow cytometry using the gating strategy shown in Supplemental Figure 4A. The results shown in Tables 1 and 2 and in Figure 3, A and B, revealed that the proportion of CD34^+^ cells positive for NKG2D-Ls was markedly higher in patients with FA (36.55% ± 4.23%) than in HDs (5.48% ± 1.34%). Additionally, the levels of NKG2D-Ls were much higher in CD34^+^ cells from patients with FA than in those from HDs (see NKG2D-L expression in representative analyses from Figure 3A). In 2 patients with FA, we were able to demonstrate an elevated proportion of CD34^+^ cells with high levels of NKG2D-Ls in periodic BM aspirations spaced 6 months to 2 years apart, revealing that the expression of NKG2D-Ls in FA HSPCs was not a sporadic or transient phenomenon (Table 1 and Figure 3B). Interestingly, analyses of NKG2D-Ls in CD34^–^ and CD34^+^ cells obtained from the same BM sample reproducibly showed that, in contrast to CD34^+^ cells, only a small proportion of CD34^–^ cells had upregulated levels of NKG2D-Ls (Supplemental Figure 4B).

We next investigated whether expression of NKG2D-Ls affects the proportion of CD34^+^ cells in BM from patients with FA. Since the highest proportion of HD BM CD34^+^ cells that expressed NKG2D-Ls was 12% (Tables 1 and 2), we classified FA BM samples into groups above and below this threshold. As shown in Figure 3C, we detected significantly lower numbers of CD34^+^ cells in the group with high expression of NKG2D-Ls. Moreover, when the proportion of CD34^+^ cells positive for NKG2D-Ls was compared with the percentage of CD34^+^ cells in BM, we found an inverse correlation (Figure 3D). These observations thus associated high expression of NKG2D-Ls with reduced numbers of CD34^+^ cells in the BM of patients with FA.

To further demonstrate the potential relationship between the expression of NKG2D-Ls in CD34^+^ cells and BMF progression, we identified the patients with a clinical diagnosis of mild or severe BMF (see last column of Table 1). Remarkably, 86.4% of FA samples (19 of 22) included in the high NKG2D-L expression group were associated with a patient diagnosis of BMF.

Taken together, these results demonstrate that, in contrast to HD CD34^+^ cells, a high proportion of BM CD34^+^ cells from patients with FA overexpressed NKG2D-Ls. Additionally, these data indicate that overexpression of NKG2D-Ls was associated with both reduced numbers of CD34^+^ cells in BM and a high incidence of BMF.

### The downregulation of FANCA in HD CD34^+^ cells increases NKG2D-L levels in a CHEK1-dependent manner.

**Figure 4:**
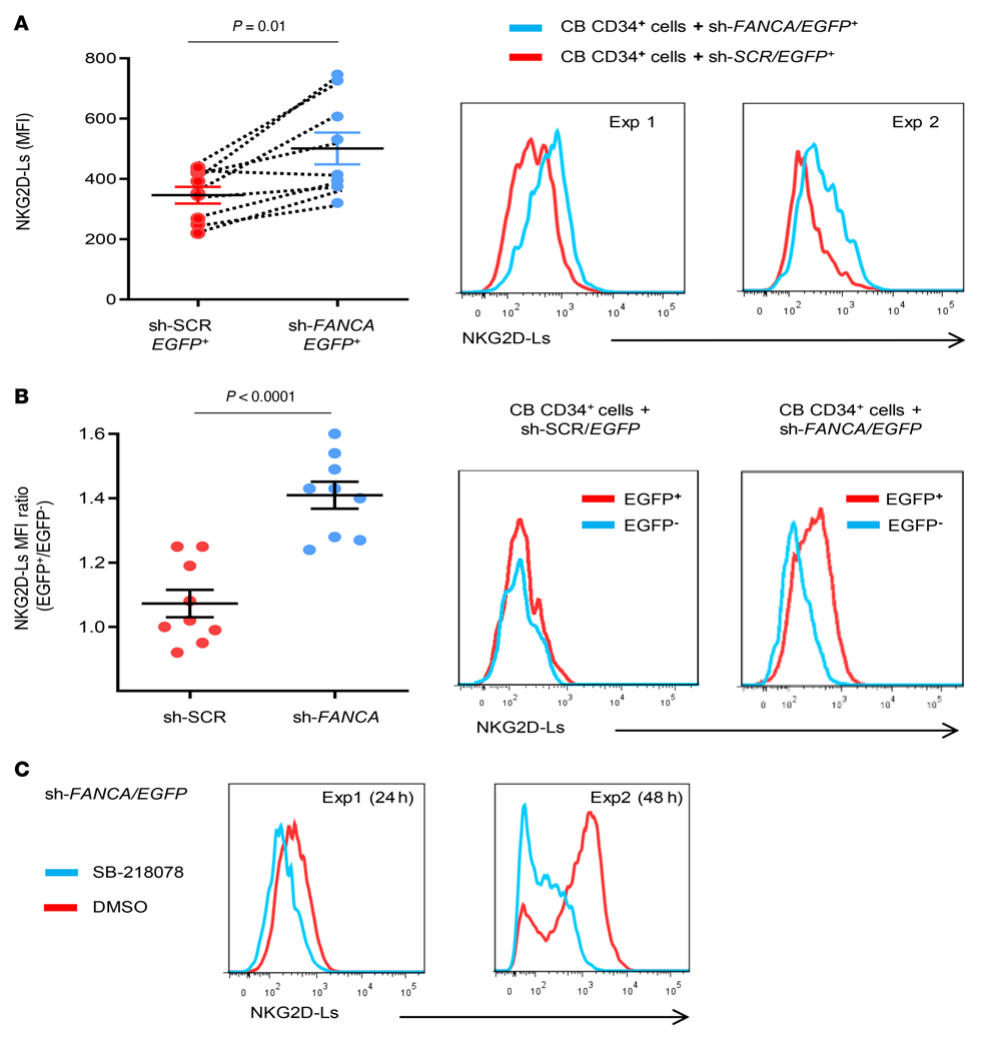
Effects of FANCA knockdown on NKG2D-L levels in HD CD34^+^ cells. (**A**) Comparative analysis of NKG2D-L in HD CB CD34^+^ cells transduced with a control shRNA (shSCR/*EGFP*) or *FANCA* shRNA (sh*FANCA/EGFP*) LVs. Left panel: NKG2D-L levels in EGFP^+^CD34^+^ cells that were transduced with either LV for 4–12 days prior to NKG2D-L analyses (*n =* 9). Right panels: Representative histograms of NKG2D-L levels in CD34^+^EGFP^+^ cells transduced with either LV. NKG2D-Ls were quantified 7 days (Exp 1) or 12 days (Exp 2) after transduction. (**B**) Left panel shows comparative analyses of NKG2D-L MFI ratios between EGFP^+^ and EGFP^–^ cells in samples from the same culture as in **A** (*n =* 9). Right panels: Representative histograms showing comparative levels of NKG2D-L in EGFP^+^ versus EGFP^–^ cells after transduction with shSCR/*EGFP* or sh*FANCA/EGFP* LVs. (**C**) Seven days after transduction, FA-like CD34^+^ cells were incubated with a CHEK-1 inhibitor or DMSO vehicle, and NKG2D-L levels were analyzed in EGFP^+^ cells. Histograms show NKG2D-L levels in 2 independent experiments after incubation with a CHEK-1 inhibitor for 24 hours (Exp 1) or 48 hours (Exp 2). Differences in **A** were determined by paired *t* test. Differences in **B** were determined by Mann-Whitney *U* test. Mean values ± SEM are shown.

To prove the relevance of a functional FA/BRCA pathway for the HSPC NKG2D-L levels, CD34^+^ cells from HD cord blood (CB) samples were transduced with lentiviral vectors that expressed either an anti-*FANCA* shRNA together with an *EGFP* marker gene (sh*FANCA/EGFP* LV; see Methods) or a scrambled shRNA and *EGFP* (sh*SCR/EGFP* LV). Four to 12 days after transduction, we analyzed the levels of NKG2D-Ls in EGFP^+^ cells by flow cytometry and detected higher levels of NKG2D-Ls in CD34^+^ cells that had been transduced with the sh*FANCA/EGFP* LV (FA-like CD34^+^ cells) compared with samples transduced with the shSCR/*EGFP* LV (see individual data and representative analyses in Figure 4A). Similarly, when ratios of NKG2D-L MFIs between EGFP^+^ and EGFP^–^ cells from the same cultures were compared, we observed significantly higher ratios in the FA-like cells (MFI ratio: 1.41) compared with ratios in the control group (MFI ratio: 1.07) (Figure 4B).

Since it has been previously reported that the DNA damage response (DDR) can lead to increased levels of NKG2D-Ls in a process dependent on ataxia telangiectasia and Rad3-related protein/checkpoint kinase 1 (ATR/CHEK-1) activation (30, 31), we analyzed whether the upregulation of NKG2D-Ls in FA-like HSPCs was dependent on ATR/CHEK-1 activation. With this aim in mind, we conducted 2 experiments in which FA-like CD34^+^ cells were incubated with a specific inhibitor of CHEK-1 (SB-2118078) that prevents the MMC-mediated accumulation of FA cells in the G_2_/M phase of the cell cycle and also the phosphorylation of CHEK-1 in FA cells (Supplemental Figure 5, A and B, respectively). Remarkably, the CHEK-1 inhibitor downregulated NKG2D-L levels in FA-like CD34^+^ cells (Figure 4C), further supporting the implication of DDR in the upregulation of NKG2D-Ls in FA HSPCS

### An increased proportion of NK and CD8^+^ T cells is observed in BM from patients with FA.

**Figure 5:**
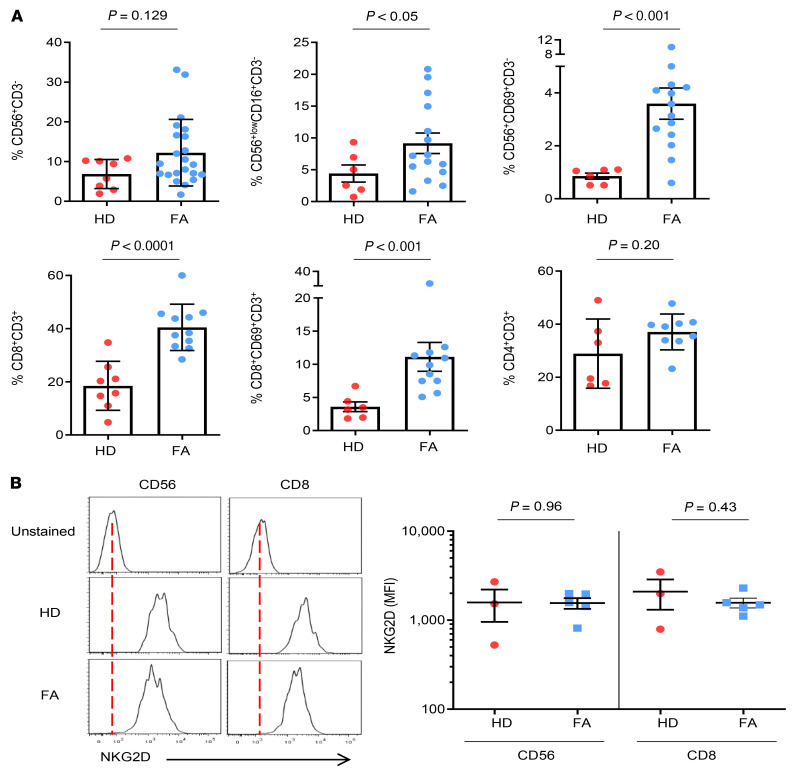
Comparative analysis of BM NK and T cells from HDs and patients with FA. (**A**) Percentage of NK cells (CD56^+^CD3^–^; FA *n =* 22, HD *n =* 8) and of NK cells with strong cytotoxic capacity (CD56^+/lo^CD16^+^CD3^–^; FA *n =* 15, HD *n =* 6) and activated NK cells (CD56^+^CD69^+^CD3^–^; FA *n =* 14, HD *n =* 6) in mononuclear BM cells from HDs and patients with FA. Lower panels show the corresponding percentages of BM cytotoxic T cells (CD8^+^CD3^+^; FA *n =* 11, HD *n =* 8), activated cytotoxic T cells (CD8^+^CD69^+^CD3^+^; FA *n =* 11, HD *n =* 6), and Th cells (CD4^+^CD3^+^; FA *n =* 6, HD *n =* 9). To compare data between HD and FA samples, an unpaired *t* test was used for comparisons between CD8^+^CD3^+^ cells, with Welch’s correction for CD56^+lo^CD16^+^CD3^–^ and CD4^+^CD3^+^ cells, and the Mann-Whitney *U* test was used for comparisons between CD56^+^CD3^–^, CD56^+^CD69^+^CD3^–^, and CD8^+^CD69^+^CD3^+^ cells. (**B**) Left panels show representative histograms of NKG2D receptor levels in NK and CD8^+^ T cells from HDs and patients with FA. Right panels show comparative analyses of NKG2D receptor expression in BM samples from HDs and patients with FA (*n =* 3). An unpaired *t* test was used for comparisons of NKG2D receptor expression between HD and FA samples in CD56^+^ and CD8^+^ cell populations. Mean values ± SEM are shown in all panels.

Since NK and T cells equipped with NKG2D receptors could mediate deleterious effects in NKG2D-L–expressing HSPCs, we first determined the proportion and activation phenotype of NK and CD8^+^ T cells in BM from HDs and patients with FA. As shown in Figure 5A, a moderate though nonsignificant increase in the proportion of NK cells (CD56^+^CD3^–^) was observed in BM samples from patients with FA compared with samples from HDs. Differences became significant for NK cells with a high capacity to mediate natural and antibody-dependent cellular cytotoxicity (CD56^+lo^CD16^+hi^CD3^–^) (32), and also for activated and tissue-resident NK cells (CD56^+^CD69^+^CD3^–^) (33). We also detected significantly higher percentages of both CD8^+^CD3^+^ and activated CD8^+^CD69^+^CD3^+^ cytotoxic T cells in BM samples from patients with FA compared with those from HDs, whereas no differences were observed for CD4^+^CD3^+^ cells (Figure 5A). Finally, we confirmed the similar expression levels of the NKG2D receptor on CD56^+^ T cells as well as on CD8^+^ T cells from patients with FA and HDs (Figure 5B).

These results demonstrated an increased percentage of activated NKG2D^+^ cytotoxic immune cells in BM from patients with FA, which might generate detrimental effects in NKG2D-L–expressing HSPCs residing in this tissue.

### Inhibition of the clonogenic potential of FA patients’ HSPCs mediated by NKG2D–NKG2D-L interactions.

**Figure 6:**
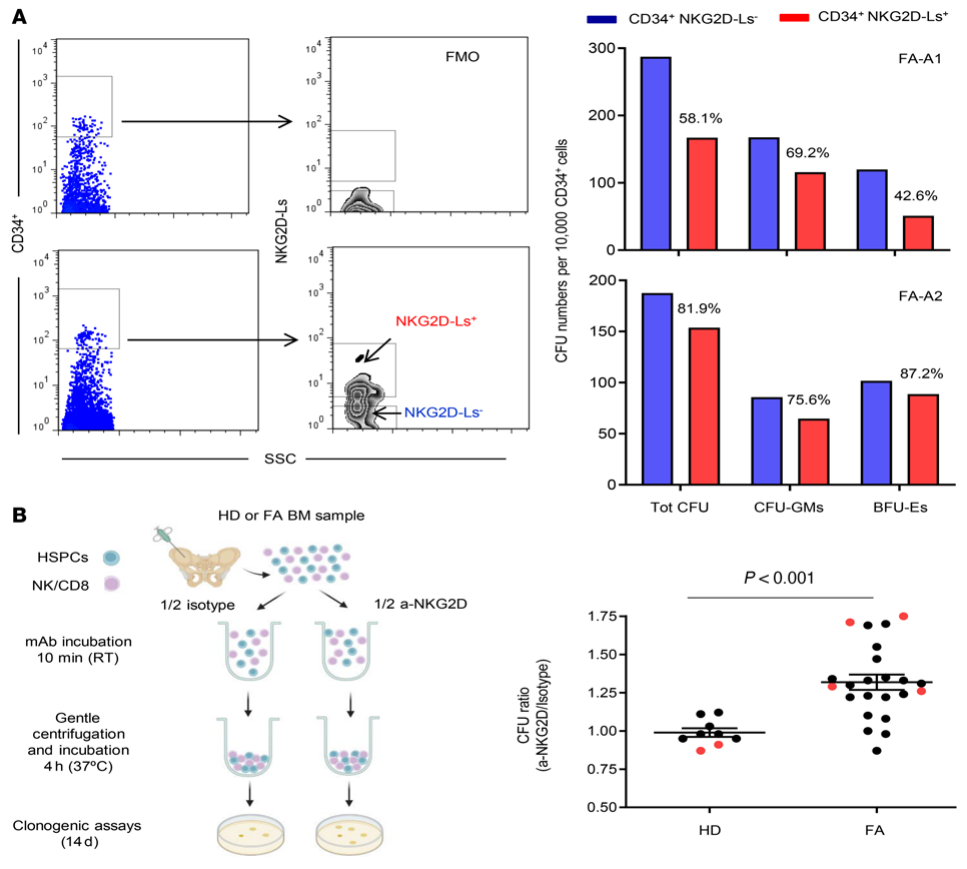
Clonogenic potential of NKG2D-L^+^ and NKG2D-L^–^ FA CD34^+^ cells and NKG2D receptor blockade effects. (**A**) Analysis of the clonogenic potential of NKG2D-L–expressing and nonexpressing BM CD34^+^ cells from 2 patients with FA. Left panels show the cell-sorting strategy used to determine the clonogenic potential of NKG2D-L^+^ and NKG2D-L^–^ CD34^+^ cell fractions. FMO corresponds to the PE fluorochrome used for the sorting of NKG2D-L^+^ and NKG2D-L^–^ cells. Right graphs show the colony numbers generated by NKG2D-L^+^ and NKG2D-L^–^ CD34^+^ cells from patients with FA. The numbers above the red bars indicate the percentage of colonies generated by NKG2D-L^+^ compared with NKG2D-L^–^ CD34^+^ cells. (**B**) Effects mediated by NKG2D receptor blockade on the clonogenic potential of BM samples from HDs and patients with FA. Left panel shows the experimental protocol used to evaluate the clonogenic effects mediated by inhibition of the NKG2D receptor. Right panel shows the ratio between the number of hematopoietic colonies generated by HD (*n =* 9) or FA (*n =* 23) BM samples after incubation with an anti-NKG2D–blocking antibody (a-NKG2D) or an isotype control. Black data points represent colony numbers generated in experiments performed with mononuclear BM cells. Some experiments (red data points) were performed using purified BM CD34^+^ cells (as target cells) and purified BM NK cells (as effector cells) from the same donor (effector/target cell ratio of 10:1). Mean values ± SEM are shown. An unpaired *t* test was used to compare mean values. Total colony numbers are shown in Supplemental Figure 7.

In this set of experiments, we determined whether FA CD34^+^ cells expressing NKG2D-Ls consisted of cells already destined to die, even in the absence of interactions with immune cells. First, analyses of annexin V expression in BM samples from 4 patients with FA revealed that 43%–73% of FA CD34^+^NKG2D-L^+^ cells were annexin V^–^, indicating that a high proportion of these cells did not show signs of apoptosis (Supplemental Figure 6). To investigate the proliferation integrity of CD34^+^ cells that expressed NKG2D-Ls, in subsequent experiments, we determined the clonogenic potential of purified NKG2D-L^+^ and NKG2D-L^–^ CD34^+^ cells from 2 patients with FA. Although sorted NKG2D-L^–^ cells from both patients generated higher colony numbers compared with NKG2D-L^+^ cells, we found that a number of colonies were also generated by NKG2D-L^+^ cells (range: 42%–81.9% of colony numbers generated by NKG2D-L^–^ cells; Figure 6A). These results thus confirmed the proliferation integrity of an important number of NKG2D-L-expressing FA CD34^+^ cells.

To evaluate the effect of NKG2D–NKG2D-L interactions between cells of the immune system (such as NK and CD8^+^ T cells) and HSPCs, we conducted in vitro NKG2D–blocking assays with BM samples from patients with FA and HDs. In these studies, mononuclear BM cells from each donor, containing both the immune effector cells and the target HSPCs, were incubated either with an anti-NKG2D–blocking antibody (a-NKG2D) or with a matching isotype control. Thereafter, we performed clonogenic assays to assess the effects mediated by NKG2D receptor blockage on colony numbers (see the experimental protocol in Figure 6B). In the case of HD samples, colony numbers generated in the presence of the a-NKG2D mAb and the isotype control were very similar, with a ratio of colony numbers of 0.98 ± 0.02. Nevertheless, when FA BM samples were tested in the same manner, this ratio was significantly increased to 1.31 ± 0.04 (*P* < 0.001) (see ratios in Figure 6B and total colony numbers in Supplemental Figure 7).

Since NKG2D is a key activation receptor for NK cells, we conducted additional studies with purified CD56^+^ cells to evaluate the inhibitory effects of these cells on purified CD34^+^ cells obtained from the same HD or FA BM sample. Similar to the effects observed with nonpurified samples, we found that the numbers of colonies were increased when the a-NKG2D mAb was added to the cocultures of purified CD34^+^ and CD56^+^ cells from patients with FA. Importantly, increases in the number of colonies did not occur when purified samples from HDs were used (Figure 6B and Supplemental Figure 7). Taken together, these in vitro experiments demonstrate that immune cells present in the BM of patients with FA, including purified NK cells, induced an NKG2D-mediated inhibitory effect against autologous HSPCs.

### Blockage of NKG2D–NKG2D-L interactions ameliorates anemia in a BMF FA mouse model.

**Figure 7:**
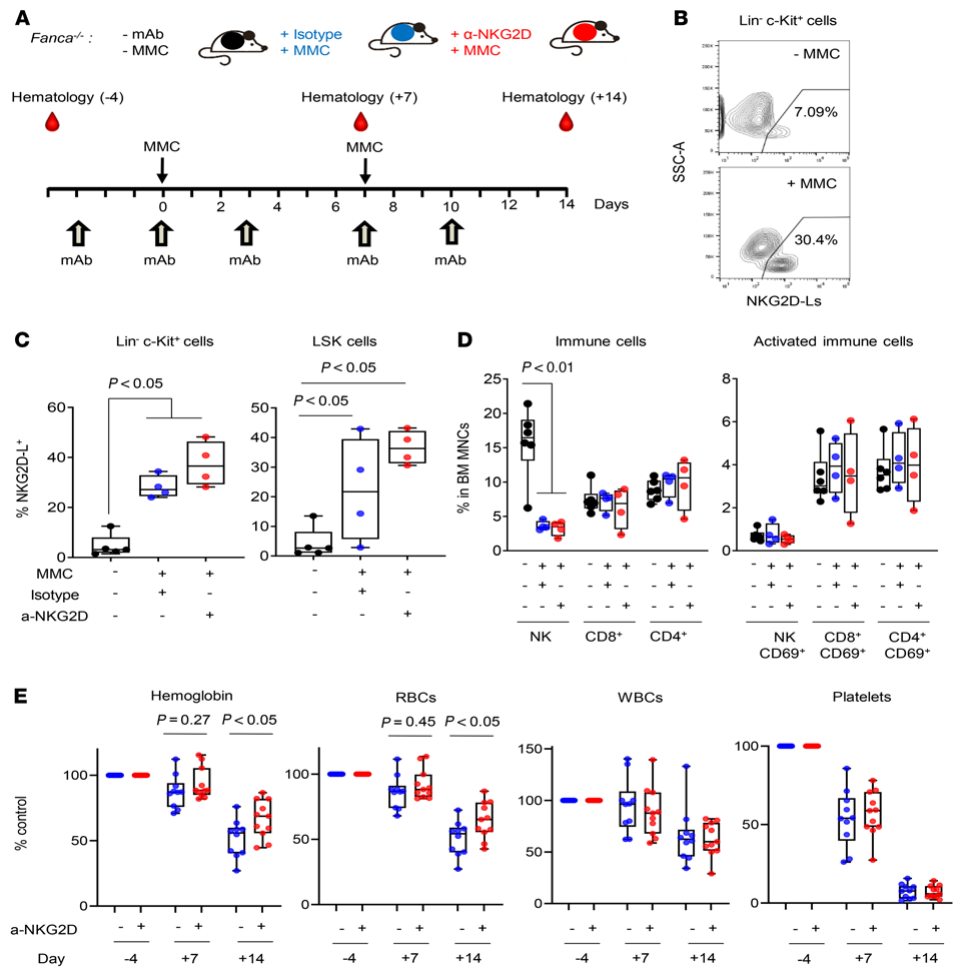
Implications of NKG2D–NKG2D-L interactions in a *Fanca^–/–^* mouse BMF model. (**A**) Experimental protocol used for evaluating the effect of NKG2D–NKG2D-L interactions in the BMF induced by MMC in *Fanca^–/–^* mice. Mice were periodically treated with an isotype control or an anti-NKG2D mAb, prior to and after initiating MMC treatment (2 doses of 0.3 mg/kg). PB samples from *Fanca^–/–^* mice were obtained prior to and after treatment. (**B**) Representative flow cytometric analysis showing expression of NKG2D-Ls in Lin^–^c-Kit^+^ cells from *Fanca^–/–^* mice treated with MMC compared with expression in untreated mice. (**C**) Proportion of Lin^–^c-Kit^+^ cells and LSK cells positive for NKG2D-Ls at the end of the experimental protocol (day 14) in untreated or MMC-treated *Fanca^–/–^* mice receiving the isotype control or the anti-NKG2D antibody. (**D**) Proportion of NK cells and CD8^+^ and CD4^+^ T cells, and their respective activated subpopulations in mononuclear BM cells from *Fanca^–/–^* mice treated as in **C**. (**E**) Comparative analysis of PB cell parameters in MMC-treated *Fanca^–/–^* mice that received the isotype control (blue dots) or the anti-NKG2D mAB (red dots). Each dot shows changes in PB parameters on days 7 and 14 after MMC treatment with respect to the values determined on day –4 (prior to starting mAb and MMC treatments), which were considered as 100%. The mean PB values corresponding to the isotype control– and the anti-NKG2D–treated groups on day –4 were, respectively, hemoglobin: 14.53 and 13.38 g/dL; RBCs: 10.37 and 9.32 × 10^6^ cells/μL; WBCs: 4.64 and 5.08 × 10^3^ cells/μL; platelets: 1002 and 914 × 10^3^ cells/μL. The Mann-Whitney *U* test was used to compare day 7 mean values and an unpaired *t* test to compare day 14 mean values according to the respective data distributions. Whiskers represent the minimum and maximum values, the lower and upper box edges correspond to the 25th and 75th percentiles, respectively, and the lines within the boxes correspond to the median.

In a final set of experiments in a BMF FA mouse model, we sought to determine whether the blockage of NKG2D–NKG2D-L interactions is associated with protective hematopoietic effects. Because *Fanca^–/–^* mice do not develop spontaneous BMF, we treated these mice with MMC as described previously for *Fancc*^–/–^ mice (34). Concomitant to the administration of MMC, mice were treated with a blocking a-NKG2D mAb or with an isotype control (Figure 7A). As shown in representative analyses from Figure 7, B and C, MMC markedly increased the proportion of hematopoietic progenitors (Lin^–^c-Kit^+^) and primitive hematopoietic stem cells (HSCs) (Lin^–^Sca1^+^c-Kit^+^ HSCs; LSK cells) expressing NKG2D-Ls. Administration of the a-NKG2D mAb further increased the proportion of HSPCs expressing NKG2D-Ls, although the differences did not reach statistical significance. In all experimental conditions, we confirmed the presence of NK cells and CD8^+^ and CD4^+^ T cells in BM from *Fanca^–/–^* mice (Figure 7D). In these analyses, we also observed that, although the overall proportion of NK cells in BM samples was decreased after MMC treatment, the percentage of NK cells and CD8^+^ and CD4^+^ T cells in MMC-treated mice was similar in the a-NKG2D and isotype treatment groups.

When hematological parameters were analyzed (Figure 7E), we observed higher hemoglobin values and numbers of RBCs in *Fanca^–/–^* mice treated with the a-NKG2D mAb compared with isotype-treated control mice. These values reached statistical significance on day 14 after MMC treatment. In contrast, no differences between the 2 groups of mice were observed when the numbers of WBCs and platelets were considered.

Overall, this final set of in vivo experiments demonstrates that the blockage of NKG2D–NKG2D-L interactions mediated a protective effect in the erythrocyte lineage in a BMF FA mouse model.

## Discussion

Previous studies have shown that DNA damage generated by exogenous or endogenous toxic sources, and even the replicative stress of FA cells, can induce DDR signaling, which activates ATR and ataxia telangiectasia mutated (ATM) cellular sensors as well as downstream proteins such as CHEK-1 and TP53 (35, 36). Given previous evidence showing that the DDR upregulates the expression of NKG2D-Ls, and taking into account that NKG2D-L–expressing cells could be recognized by cells of the immune system (25, 27, 30, 31), we hypothesized that FA cells could be prone to overexpress NKG2D-Ls, and also that this upregulation on FA HSCs could play a relevant role in the etiology of BMF progression in patients with FA.

Our first studies in untreated fibroblasts and PB T cells showed that NKG2D-L levels were significantly higher in FA cells than in HD cells, and that either MMC or formaldehyde — a physiological source inducing endogenous DNA damage (37) — significantly increases NKG2D-L levels in FA cells. The direct involvement of the FA pathway in the regulation of NKG2D-L levels was confirmed in experiments in which the mere complementation of the FA genetic defect prevented the upregulation of these molecules, showing the relevance of this DNA repair pathway in the modulation of NKG2D-Ls.

Although all NKG2D-Ls interact with the same NKG2D receptor (28), we observed that increased levels of NKG2D-Ls in FA fibroblasts were mainly due to elevated levels of MICA as well as of MICB, ULBP3, and ULBP4. Because of the complexity of the regulatory mechanisms involved in modulating NKG2D-Ls, including transcriptional, posttranscriptional, and posttranslational levels (see review in ref. 38), specific studies should be performed to clarify the mechanisms accounting for the upregulated expression of the individual NKG2D-Ls in different FA cell types.

Remarkably, analyses conducted in fresh BM samples revealed that, in contrast to HD CD34^+^ cells, a large proportion of CD34^+^ cells from patients with FA expressed high levels of NKG2D-Ls. Nevertheless, this upregulation was not observed in CD34^–^ cells obtained from the same BM sample, suggesting that, as shown in different cancer cell types (39), the upregulation of NKG2D-Ls is most evident in cells of the stem cell compartment, in this particular case, in FA HSPCs. Even more relevant was our observation of an inverse correlation between the proportion of CD34^+^ cells in FA patients’ BM and the proportion of CD34^+^ cells expressing NKG2D-Ls, which strongly points to the negative impact of NKG2D-L expression in FA HSPCs. These results, together with the observation that a high percentage of patients with FA with NKG2D-L–overexpressing CD34^+^ cells showed clinical signs of BMF, further support the relevance of NKG2D-L expression in the pathogenesis of BMF in FA.

Our observations showing that the downregulation of *FANCA* by lentiviral shRNA vectors (FA-like cells) upregulated NKG2D-Ls levels in HD CD34^+^ CB cells demonstrate the direct impact of the FA/BRCA pathway on NKG2D-L levels in FA HSPCs. Additionally, the fact that inhibition of CHEK-1 downregulated NKG2D-Ls in FA-like CD34^+^ cells strongly supports the hypothesis that the involvement of the DDR in increased levels of NKG2D-Ls is a direct consequence of the disruption of the FA/BRCA pathway. Interestingly, a previous study described an attenuated clinical phenotype in patients with FA with mild BMF that was associated with low levels of CHEK-1 in cells of the hematopoietic system (35). As CHEK-1 inhibition significantly reduced NKG2D-L levels in FA HSPCs, we can speculate that the low levels of CHEK-1 present in these patients with attenuated FA may prevent the overexpression of NKG2D-Ls, limiting the recognition of these primitive hematopoietic cells by immune cells. This process would account, at least in part, for the improved hematopoiesis observed in these patients.

Although CD8^+^ T cells and NK cells mainly reside in different lympho-hematopoietic tissues, both cell types can migrate to inflamed tissues (33, 40, 41). We therefore reasoned that the percentage and activation status of these NKG2D^+^ cells might be increased in the BM of patients with FA, a hypothesis that was confirmed in our comparative BM immune-phenotypic studies of HDs and patients with FA. Whether CD8^+^ T cells and NK cells were activated in situ or recruited as already preactivated cells is currently unknown.

Although it could be speculated that NKG2D-L–expressing HSPCs are already destined to die, our results indicate that this is not the case for a significant proportion of these cells. This conclusion proceeds from our experiments showing that a proportion of FA CD34^+^ NKG2D-L^+^ cells are characterized by negative annexin V staining and retain a significant clonogenic capacity. Moreover, NKG2D-blocking experiments with either purified or nonpurified CD34^+^ and NK cells demonstrated that, at least in vitro, immune BM cells from patients with FA exert NKG2D-mediated inhibitory effects on the clonogenic potential of FA HSPCs, an effect that was not observed when HD BM cells were used.

Since NKG2D not only constitutes a strong activating receptor of NK cells, but is also a coreceptor in CD8^+^ cells (19), we cannot rule out the possibility that FA CD8^+^ cells might also exert some additional effect against FA HSPCs. However, it seems unlikely that T cells are involved in this process, as multiple studies of T cell immunosuppression have only shown hematologic improvements in acquired BMF syndromes, but not in inherited ones such as FA (42).

In our final set of experiments, we investigated the hematopoietic effects that NKG2D–NKG2D-L interactions might have in vivo. In these studies, we used a BMF *Fanca^–/–^* mouse model based on MMC administration, as previously described in *Fancc^–/–^* mice (34). Remarkably, our data showed that the inhibition of NKG2D–NKG2D-L interactions enhanced hemoglobin values and RBC numbers in this BMF mouse model. Strikingly, it was also in the erythrocyte lineage in which we first noted the improvement as a result of HSC gene therapy in patients with FA-A (3).

Currently, suppressive NKG2D-mediated processes against HSPCs have only been associated with the BMF that takes place in patients with acquired aplastic anemias (15, 16). Our study provides evidence of HSPC-inhibitory effects mediated by NKG2D–NKG2D-L interactions in an inherited BMF syndrome, in particular in FA. We propose that the DDR generated by exogenous or endogenous damaging agents and/or by the replicative stress of FA HSPCs would result in different cellular alterations leading to NKG2D-L overexpression in these cells. Once recognized by NKG2D^+^ effector immune cells, these would exert cytotoxic effects through perforins and granzymes and/or the secretion of proinflammatory cytokines such as TNF-α and IFN-γ. These immune mechanisms would thus contribute to the onset or amplification of an inflammatory environment and increased HSPC damage in FA BM.

Overall, our study suggests an unexpected role of the immune system in the progressive BMF that is characteristic of patients with FA. Detailed follow-up studies in large series of patients with FA would indicate whether the overexpression of NKG2D-Ls in HSPCs could be used as an early event predicting BMF in patients with FA. Additionally, our results present alternative therapeutic options to delay BMF onset in patients with FA, based on the pharmacological inhibition of NKG2D–NKG2D-Ls interactions. Nevertheless, taking into account the cancer-prone nature of FA cells and the paramount role of these interactions in the elimination of tumor cells, these strategies would need to be carefully evaluated in patients with FA.

## Methods

### Samples from patients with FA and HDs.

All patients with FA had a positive chromosomal breakage test, and most of them corresponded to the FA-A complementation group (Table 1). Patients with FA with somatic mosaicism were excluded from this study. In all instances, fresh BM samples were analyzed within the first 48 hours after BM aspiration. Patients with BMF at the moment of the analysis were defined as those with hypocellular BM, and 2 of the following PB criteria: hemoglobin <10 g/dL; neutrophils <1.5 × 10^9^/L; platelets <50 × 10^9^/L. Patients with only 1 affected PB lineage were defined as having mild BMF. The percentage of aberrant T cells in the diepoxybutane (DEB) breakage test corresponds to analyses performed at diagnosis. Umbilical CB samples to obtain HD CD34^+^ cells were provided by the Centro de Transfusión de la Comunidad de Madrid.

### Cultures of human fibroblasts and PB T cells.

All primary cells were generated at the CIEMAT (Madrid, Spain). Primary skin fibroblasts were cultured in DMEM plus GlutaMAX (Gibco, Thermo Fisher Scientific) supplemented with 20% FBS (HyClone). Mononuclear PB cells were obtained after Ficoll-Paque PLUS (GE Healthcare) gradient centrifugation of PB samples. Cells were washed in PBS and cultured in nontreated plates coated with purified anti–human CD3 (catalog 555728) and purified anti–human CD28 (555356) mAbs (both from BD Biosciences) with IMDM plus GlutaMAX (Gibco, Thermo Fisher Scientific) with 20% FBS as previously reported to activate T cell proliferation (43).

### Hydroxyethyl starch sedimentation of BM samples and purification of CD34^+^ cells from CB.

Fresh heparinized BM aspirates from HDs and patients with FA were subjected to hydroxyethyl starch sedimentation (HES) to remove erythrocytes and maximize the number of collected leukocytes (44). Briefly, BM samples were mixed with 6% HES (Grifols) and left undisturbed for 4 hours at room temperature, allowing RBCs to sediment by gravity. Cells were washed in PBS, and the remaining RBCs were lysed with ammonium chloride solution (8.3 g/L ClNH_4_, 1 g/L KHCO_3_, and 2 mL 0.5 M EDTA). After 3 minutes, cells were again washed in PBS and resuspended in PBA (PBS and 0.1% BSA). CD34^+^ cells from HD CB were isolated after Ficoll-Paque PLUS gradient centrifugation and subsequent immunomagnetic cell sorting (microBead kit, human, Miltenyi Biotec) according to the manufacturer’s instructions.

### Flow cytometry and sorting of cells from human BM samples.

To analyze the expression of all known NKG2D-Ls in HD and FA cells, a mixture of the anti–human NKG2D-L antibodies MICA-PE (clone, 159227), MICB-PE (clone, 236511), ULBP1-PE (clone, 170818), ULBP2,5,6-PE (clone, 165903), ULBP3-PE (clone, 166510), and ULBP4-PE (clone, 709116) (all from R&D Systems) was prepared. Analyses of individual NKG2D-Ls in fibroblasts were also performed. To analyze the expression of NKG2D-Ls in HSPCs, cells were stained with an anti–CD34-FITC antibody (clone 8G12) from BD Biosciences, together with the mixture of NKG2D-L antibodies. Prior to cell labeling with the corresponding antibodies, BM cells were incubated with an FcR-blocking reagent (Miltenyi Biotec) to prevent nonspecific binding. At least 500,000 mononuclear cells from BM were analyzed per sample to quantify the level of NKG2D-Ls in CD34^+^ cells. CD34-APC (clone 581) from BD Biosciences and Annexin V-FITC/IP from BD Pharmingen were used to analyze early apoptosis in CD34^+^ cells labeled with the mixture of NKG2D-L-PE antibodies. The percentage of CD34^+^ cells in BM aspirates was quantified following the International Society of Hematotherapy and Graft Engineering (ISHAGE) guidelines for CD34^+^ cell determination by flow cytometry using CD34-PE (clone 8G12) from BD Biosciences and CD45-FITC (clone J33) from Immunotech. To study the immune phenotype in BM, cells were stained with antibodies against the following surface markers: CD3-APC (clone UCHT1) from BioLegend, CD56-FITC (clone NCAM16.2) from BD Biosciences, CD8-FITC (clone B9.11) from Beckman Coulter, CD4-FITC (clone 13B8.2) from Beckman Coulter, CD69-PE (clone FN50) from BioLegend, and CD16-PCy5 (clone 3G8) from BD Biosciences. In all flow cytometric studies, DAPI was used to discard dead cells in the analysis. Flow cytometry was performed on an LSR Fortessa cytometer (BD Biosciences), and data were analyzed using FlowJo software, version 10.8.1 (Tree Star).

Antibodies CD56-PE (clone N90) form Beckman Coulter and CD34-APC (clone 581) from BD Biosciences were used for the sorting of BM NK cell populations and HSPCs, respectively. CD34-APC (clone 581) and CD45-FITC (clone J33) from Beckman Coulter, and the mixture of NKG2D-Ls–PE antibodies were used to discriminate BM CD34^+^NKG2D-L^+^ and CD34^+^NKG2D-L^–^ cells in a BD Influx Cell Sorter. A fluorescence minus one (FMO) control stained with anti–CD34-APC and anti–CD45-FITC antibodies was used to set the gating of CD34^+^NKG2D-L^+^ and CD34^+^NKG2D-L^–^ cells. Sorted cells were cultured in methylcellulose-based medium (see below).

### Colony-forming cell assays.

Clonogenic assays were performed using methylcellulose-based medium (MethoCult H4434, STEMCELL Technologies). Fourteen days after seeding of the cells, hematopoietic colonies (total colonies and CFU-GM and BFU-E colonies) were scored under an inverted phase-contrast microscope at ×40 (Nikon Diaphot). An appropriate number of total or purified BM cells were seeded to facilitate the scoring of 10–100 colonies per plate.

### NKG2D-Ls and phosphorylated H2AX immunofluorescence studies.

For the NKG2D-L staining of HD and FA fibroblasts, cells were seeded in polystyrene chambers (Culture Slides, Falcon) and incubated for 24 hours. Thereafter, the medium was replaced by 200 μL medium containing the NKG2D-L mAb mixture and incubated for an additional 30 minutes at 37°C. After 2 washing steps with PBS, cells were examined using an Eclipse Ts 2R microscope (Nikon; ×20 objective, ×0.5 magnification). Representative images were obtained with a DFC 7000 T camera (Leica).

To test the levels of DNA damage in uncorrected and gene-corrected FA fibroblasts, cells were cultured in CultureSlides with MMC (100 nM). Twenty-four hours after treatment, cells were fixed in 4% paraformaldehyde in PBS for 15 minutes at room temperature (RT), and after 3 washes in PBS, cells were permeabilized in 0.5% Triton X-100 (MilliporeSigma) for 5 minutes. After 3 washes, slides were blocked in TBS 0.1% NP-40 plus 10% FBS for 30 minutes and incubated with a primary antibody (anti–phosphorylated histone H2AX [Ser139], clone JBW301, MerkMillipore) diluted in the blocking buffer (1 μg/mL) at 4°C overnight. After 3 washes in TBS, cells were incubated for 1 hour at RT with the secondary fluorescent polyclonal antibody Alexa Fluor 488 F(ab′)2 goat anti–mouse IgG (H+L) (A-11017) from Invitrogen (Thermo Fisher Scientific) diluted with the blocking buffer and DAPI. Samples were visualized with an Axio Imager.M2 fluorescence microscope (Carl Zeiss; ×63 objective, ×1.4 magnification). Two hundred cells were counted, and cells with more than 10 foci per cell were scored as positive. Each data point in the figures represents data from 1 independent experiment, with the mean of 3 independent experiments also shown. Images were captured with an Axiocam 503 mono camera (Carl Zeiss) and processed with ZEN 2.3 Blue Edition software (Carl Zeiss).

### CHEK-1 inhibition and cell-cycle studies.

Fibroblasts were cultured with 33 nM and 100 nM MMC for 3 days in the presence or absence of the CHEK-1 inhibitor SB-2118078 (Calbiochem) at 1 μM. After incubation with MMC, cells were washed with PBS and fixed with 70% cold ethanol for 24 hours. Cells were washed again with PBS and incubated for 30 minutes at 37°C with 50 μg/mL propidium iodide (MilliporeSigma) and 100 μg/mL RNase A/T1 (Thermo Fisher Scientific). DNA content was analyzed by flow cytometry, avoiding cell aggregates in linear amplification.

### Transduction of human fibroblasts and T cells with RVs.

FA-A fibroblasts were transduced with RVs carrying the *FANCA* and *Neo^r^* genes (FAIN vector) to generate corrected FA-A cells. As controls, untransduced or FA-A cells transduced with RVs carrying the *FANCC* or *FANCG* genes, together with the *Neo^r^* gene (FCIN and FGIN vectors, respectively), were used (45). Transduced cells were selected with G418 at 0.7 mg/mL (Calbiochem) for 5 days and expanded prior to use. PB T cells from HDs or patients with FA-A were transduced with RVs carrying the *FANCA* and *EGFP* genes (LFAPEG) or the *FANCG* and *EGFP* genes (LGEG11) (43, 46). For the transduction of T cells, mononuclear PB cells were preincubated with immobilized anti-CD3 and anti-CD28 antibodies (see above) in 6-well, nontreated plates for 4 days. Thereafter, the cells were transduced with 2 infection cycles of the corresponding RVs (24 hours per cycle).

### MMC and formaldehyde treatments of human fibroblasts and T cells.

Fibroblasts from HDs and patients with FA-A were cultured for 3 days in the absence or presence of MMC (33 nM and 100 nM; MilliporeSigma). For formaldehyde treatments, fibroblasts were incubated for 24 hours with 25 μM and 50 μM formaldehyde (prepared from a 37% solution, proteomics grade; VWR Life Science). After incubation with MMC or formaldehyde, cells were trypsinized and stained to measure total expression of NKG2D-Ls. Activated T cells (see above) were challenged with MMC (33 nM and 100 nM) for 4 days, and NKG2D-Ls expression in DAPI^–^ cells was analyzed by flow cytometry. To evaluate the basal expression of NKG2D-Ls, the ratios of MFI values between unstained cells (–mAb) and NKG2D-L–labeled cells (+mAb) were determined.

### FANCA interference in HD CD CD34^+^ cells.

FA-like CD34^+^ cells were generated by transducing HD CB CD34^+^ cells with a sh*FANCA/EGFP* LV (47). This LV carries the sh11 and sh7 hairpin RNAs (shRNAs) that target human *FANCA* and expresses the *EGFP* marker gene. The sh*FANCA/EGFP* LV was generated in the puc2CL11Egw.sh7FA-A7 with 2 sites of interference over the *FANCA* messenger. The shSCR/*EGFP* LV was used as an interference control and contained a scrambled sequence and the *EGFP* marker gene. The functionality of the sh*FANCA/EGFP* LV has been previously demonstrated (47). HD CB CD34^+^ cells were stimulated for 2 days in IMDM plus GlutaMAX supplemented with 20% FBS and the hematopoietic growth factors stem cell factor (SCF) (30 ng/mL), thrombopoietin (TPO) (10 ng/mL), and FMS-like tyrosine kinase 3 ligand (FLT3-L) (10 ng/mL) (Miltenyi Biotec) in a humidified atmosphere at 37°C and 5% CO_2_. Cells were transduced with the shSCR/*EGFP* or sh*FANCA/EGFP* LVs using 2 infection cycles of 24 hours in plates precoated with Retronectin (Takara). Transduced cells were then incubated for 4–12 days in culture under conditions used for prestimulation. Levels of NKG2D-Ls in cells transduced with the sh*FANCA/EGFP* LV were compared with shSCR/*EGFP*-transduced cells showing similar levels of EGFP expression, and also with EGFP^–^ cells.

### CHEK-1 Western blotting.

To analyze the inhibitory activity of SB-2118078 on CHEK-1 activation, primary fibroblasts from HDs and patients with FA were cultured with the inhibitor for 24 hours (1 μM) and then treated with trypsin-EDTA (1%) and washed in PBS. To obtain cytoplasmic and nuclear protein in denaturing conditions, cells were lysed with RIPA lysis buffer (Thermo Fisher Scientific) containing 25 mM Tris-HCL, pH 7.6, 150 mM NaCl, 1% NP-40, 1% sodium deoxycholate, and 0.1% SDS with protease and phosphatase inhibitors. Lysates were immunoblotted using a rabbit mAb against phosphorylated CHEK-1 (Ser296) (Abcam, catalog ab79758), a rabbit anti-vinculin antibody (Abcam, catalog ab129002), and a goat polyclonal anti-rabbit antibody (HRP) (Abcam, ab6721), and revealed with Super Signal West Pico PLUS (Thermo Fisher Scientific).

### Incubation of the CHEK-1 inhibitor with FANCA-like CD34^+^ cells.

To analyze the role of the DNA damage sensor CHEK-1 in the expression of NKG2D-Ls in FA-like HSPCs, CD34^+^ cells were transduced with sh*FANCA/EGFP* (see above), and 7 days later, cells were treated for 1–2 additional days with the CHEK-1 inhibitor SB-218078 (Calbiochem) dissolved in DMSO at a concentration of 1 μM (48). NKG2D-Ls levels were then analyzed in CD34^+^EGFP^+^ cells and compared with control cells only treated with DMSO.

### In vitro NKG2D-blocking assays.

This blocking assay was based on methods previously reported (15). HES-fractionated BM cells from HDs and patients with FA were divided into 2 equal aliquots (range: 0.5 × 10^6^ to 1 × 10^6^ cells) and resuspended in 200 μL IMDM supplemented with 20% FBS and loaded into V-bottomed 96 MicroWell plates (Nunc). One of the aliquots was incubated with mouse IgG1 (25 μg/mL) as an isotype control (clone 11711), and the other one was incubated with the anti-NKG2D antibody (25 μg/mL) to block the NKG2D-activating receptor (clone 149810), both from R&D Systems. Cells were incubated for 10 minutes at RT and resuspended after 5 minutes. Subsequently, cells were gently centrifuged at 60*g* for 3 minutes to facilitate cell interaction at the well bottom and were then further incubated at 37°C in 5% CO_2_ for 4 hours. Cells were then collected, resuspended, and seeded in methylcellulose cultures (see experimental scheme in Figure 6). Colony numbers generated in the NKG2D-blocking group were normalized with respect to numbers corresponding to the isotype control group. Some experiments were conducted using purified CD56^+^ cells (effectors cells) and purified CD34^+^ cells (target cells) sorted by FACS (see above). In these experiments, a ratio of 10:1 (effector/target cells) was used.

### BMF model in Fanca^–/–^ mice.

Mice with a targeted disruption in the *Fanca* gene (49) were a gift of H.J. van de Vrugt (Free University Medical Center, Amsterdam, Netherlands) and were bred and maintained at our facility as previously described (50). Six- to 8-month-old female FVB-129 WT and *Fanca^–/–^* mice were maintained under high-standard conditions and routinely screened for pathogens. The induction of BMF in *Fanca^–/–^* mice was performed by i.p. administration of 2 doses of 0.3 mg/kg MMC (MilliporeSigma), spaced 7 days apart. To evaluate the relevance of NKG2D–NKG2D-L interactions to the BMF in these animals, mice were treated (i.p.) every 2–3 days with an InVivoMAb anti–mouse NKG2D antibody (CD314, clone CX5) or an isotype control InVivoMAb rat IgG1 (anti-HRP) antibody at 10 mg/kg (both from Bio X Cell). These antibodies were diluted in InVivoPure pH 7 dilution buffer (Bio X Cell) for i.p. administration. The experimental protocol used to evaluate the effect of NKG2D–NKG2D-L interactions on the BMF in *Fanca^–/–^* mice is described in Figure 7A.

To evaluate the course of the BMF, PB samples (100 μL) were periodically obtained from the tail vein and anticoagulated with EDTA (Microvette CB300, Sarstedt). PB parameters were determined in a hematological analyzer (Sysmex XN-1000). At the end of the treatment, the mice were sacrificed, and femoral BM cells were resuspended in IMDM medium (Gibco Laboratories, Thermo Fisher Scientific) supplemented with 10% FBS (Sigma Chemical, MilliporeSigma). A mixture of anti–mRae-1-PE pan-specific (FAB17582P), anti–mH-60-PE (FAB1155P), and anti–mMULT-1-PE (FAB2588P) antibodies (all from R&D Systems) was used to evaluate the expression of NKG2D-Ls in mice, as described above for the analyses of human cells. Analyses of mouse hematopoietic progenitor cells (Lin^–^c-Kit^+^) and more primitive Lin^–^Sca1^+^cKit^+^ (LSK) precursors were performed using the following mAbs for lineage-positive staining: anti–Gr-1 (clone RB6-8C5, BioLegend), anti-B220 (clone RA3-6B2, BioLegend), anti-CD3 (clone 145-2C11, BD Pharmingen), anti-CD11b (clone M1/70, eBioscience), and anti-Ter119 (clone TER119, BD Pharmingen), all with FITC. For positive staining of HSPCs, anti–Sca-1–APC–Cy7 (clone D7, BioLegend) and anti–c-Kit–PE–Cy5 (clone 2B8, eBioscience) were selected. Immune cells were analyzed using the following mAbs: anti-NK1.1 (clone PK136), anti-CD4 (clone H129.19), and anti-CD8 (clone 53–6.7), all with FITC and all from BD Pharmingen. To evaluate the immune cell activation status, these cells were stained with anti–CD69-PE (clone H1.2F3) from BD Pharmingen.

### Statistics.

Normal distribution was analyzed by the Shapiro-Wilks test. To determine statistically significant differences between independent continuous variables, a 2-tailed, paired or unpaired Student’s *t* test with Welch’s correction was applied when an equal SD was not assumed for normal distribution, and a 2-tailed, nonparametric Mann Whitney *U* test was used for non-normal distribution. For multiple comparisons, the Kruskal-Wallis test was followed by Dunn’s multiple-comparison test. Spearman’s rank correlation coefficient was used to analyze associations between 2 continuous variables. Statistical analyses were performed with GraphPad Prism, version 7.00.159 (GraphPad Software), and *P* values of less 0.05 were considered statistically significant.

### Study approval.

All experimental procedures were carried out according to Spanish and European regulations. The experimental mice procedures were approved by the committee at the CIEMAT facility in Madrid, Spain (PROEX 156.5/21, regulation no. 28079-21 A). The study involving patients with FA was approved by the Centro de Investigación Biomédica en Red de Enfermedades Raras (CIBERER) (CIBERER-16-12-2010 and CIBER 17-6-2015). Patients with FA and HDs provided written informed consent prior to participation in this study. Studies conducted with samples from HDs and patients with FA were approved by the IRB of the Hospital del Niño Jesús (Madrid, Spain) and according to the Declaration of Helsinki. Patient and HD identities were encoded to protect confidentiality. In all instances, written informed consent was obtained from the study participants.

## Author contributions

JAC conceived the study. AV, RSD, PV, AL, SN, OA, RP, JCS, JM, and J Surrallés performed the experiments. HH, provided key vectors for the study. CDH and J Sevilla provided critical samples and studied the patients. JAB directed the study and, with JAC, analyzed the data and wrote the manuscript. All authors contributed to the discussion and writing of the manuscript.

## Supplementary Material

Supplemental data

## Figures and Tables

**Table 1:**
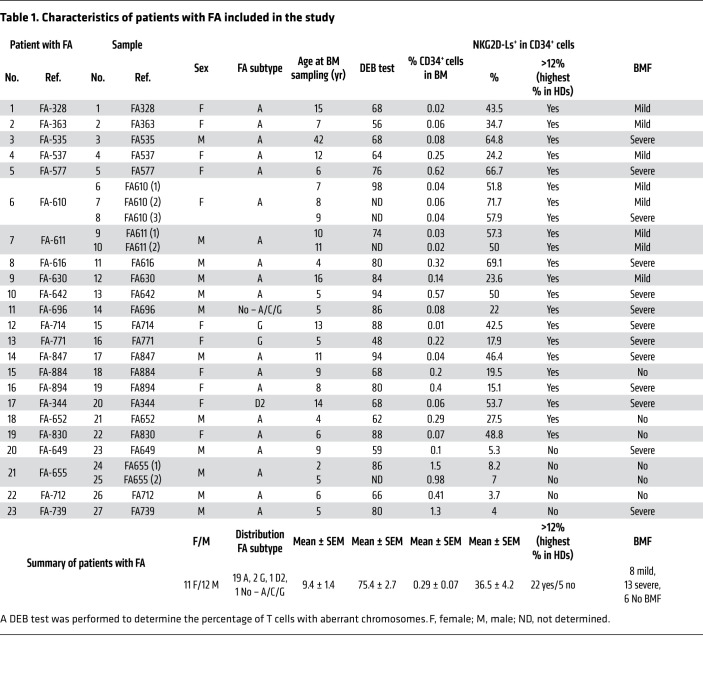
Characteristics of patients with FA included in the study

**Table 2:**
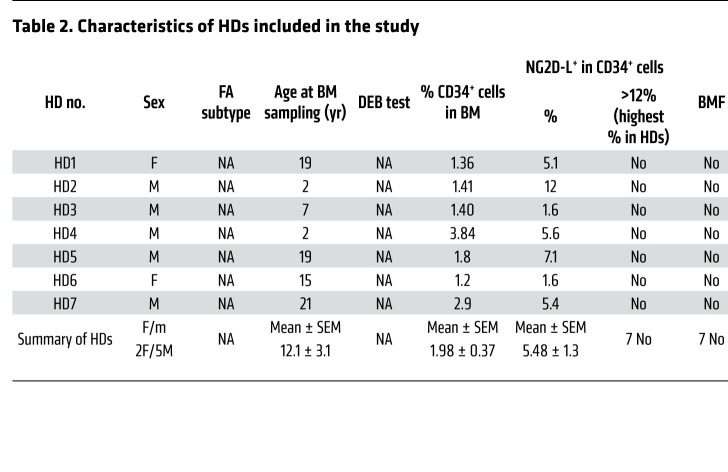
Characteristics of HDs included in the study

## Version 1 06/07/2022

In-Press Preview

## Version 2 08/01/2022

Electronic publication
