# Supplementary material for: Upregulation of NKG2D ligands impairs hematopoietic stem cell function in Fanconi anemia
Source: J Clin Invest. 2022 Aug 1;132(15):e142842. doi: 10.1172/JCI142842 (PMC9337828; doi:10.1172/JCI142842)

Supplemental Figure 1

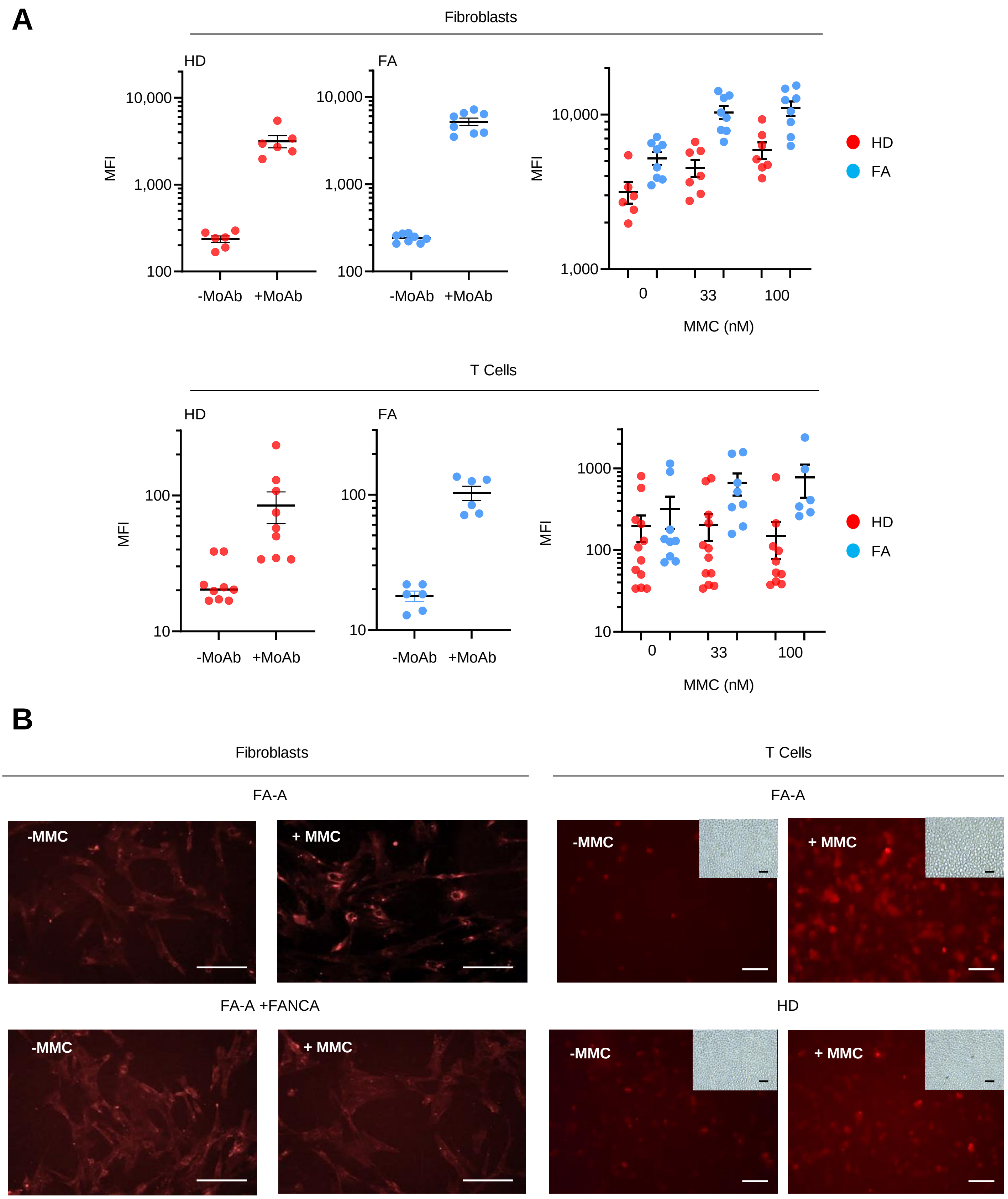

Supplemental Fig. 2

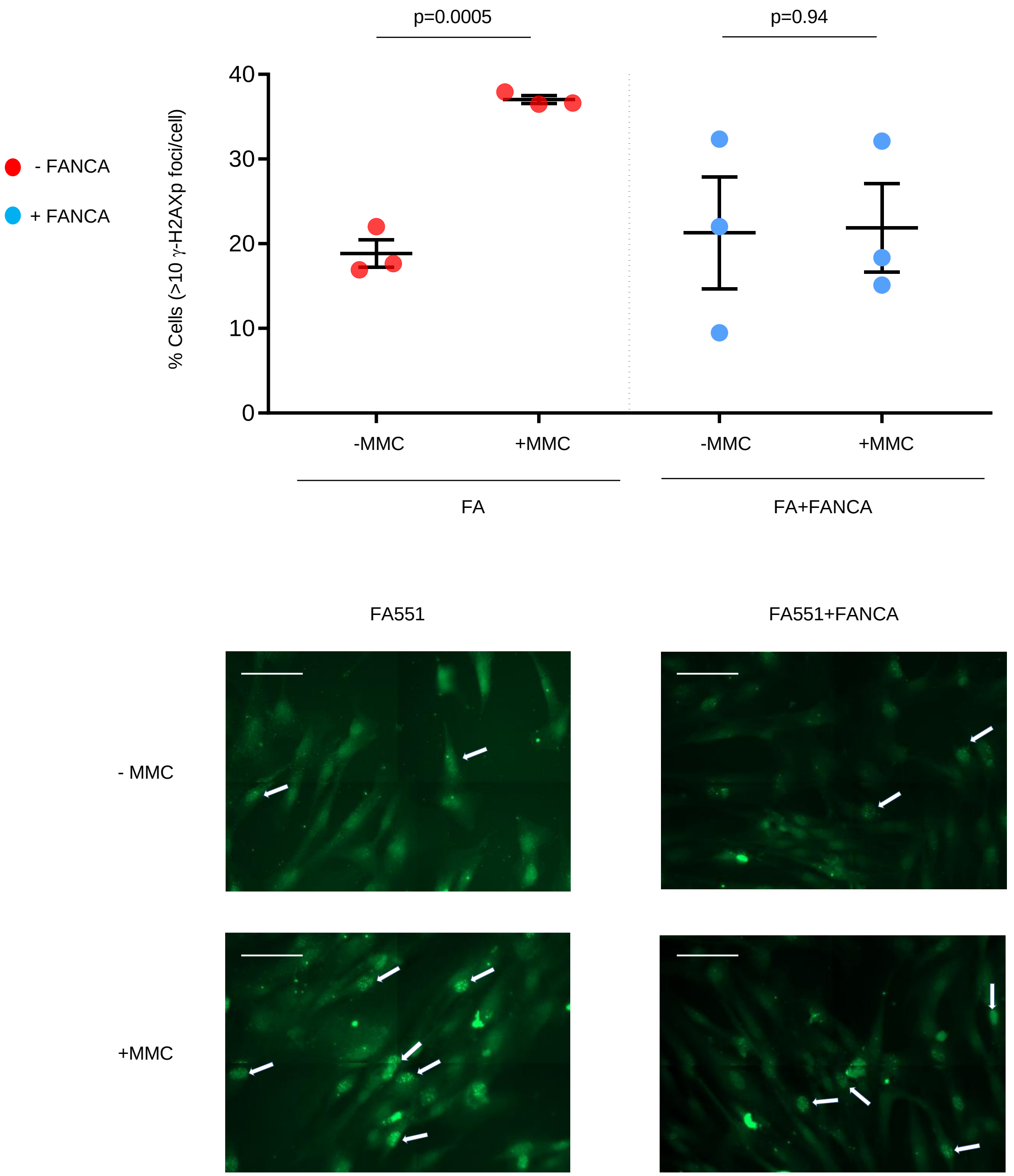

Supplemental Figure 3

A

Combined
NKG2D-L MoAbs

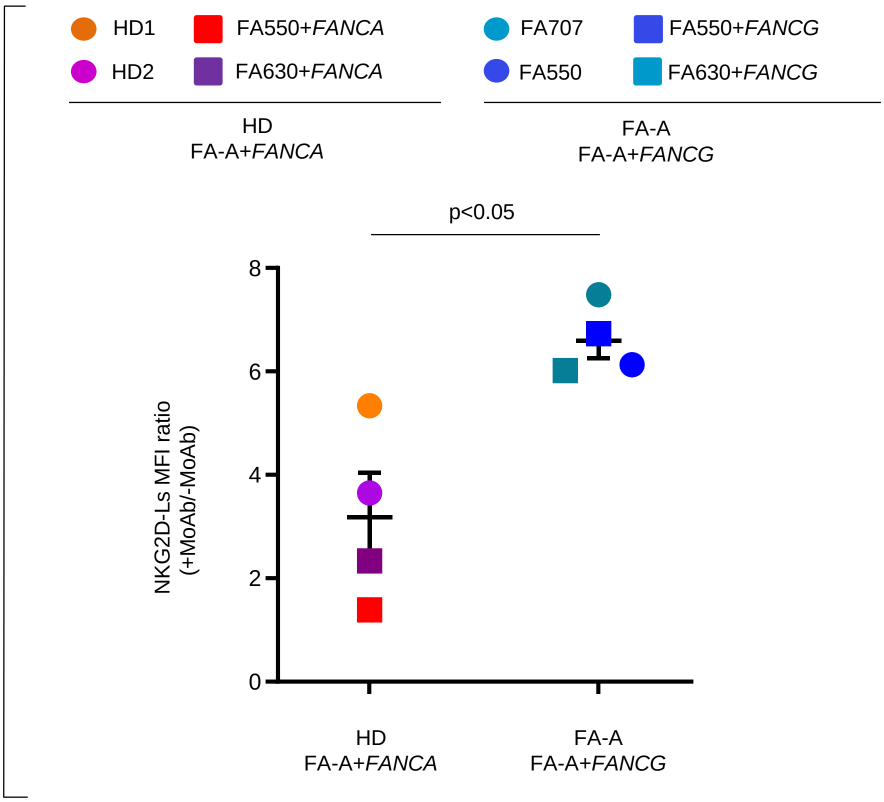

B

Individualized
NKG2D-L MoAbs

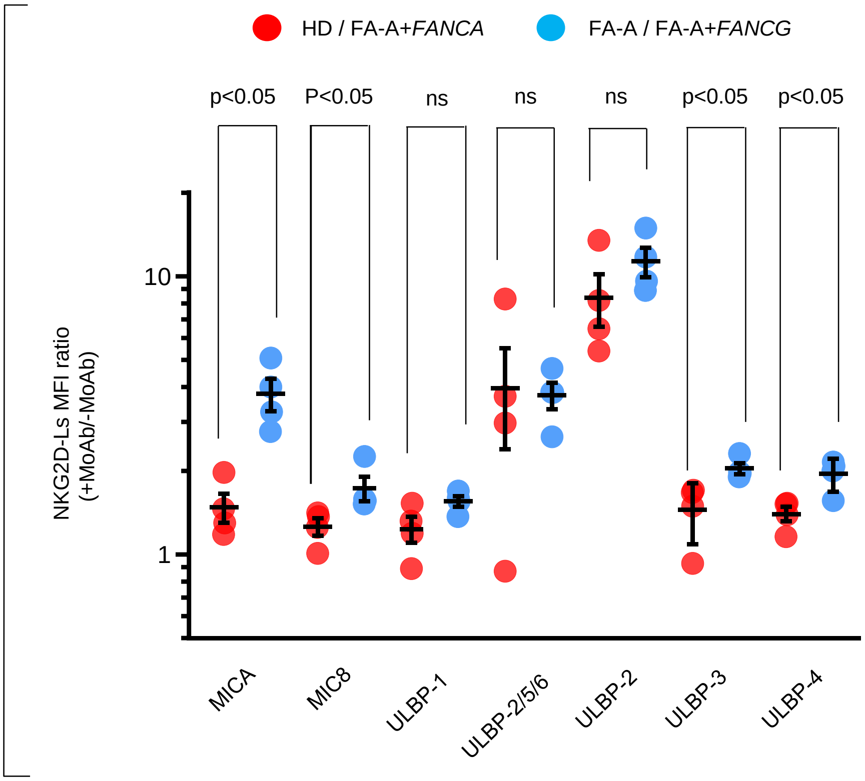

Supplemental Figure 4

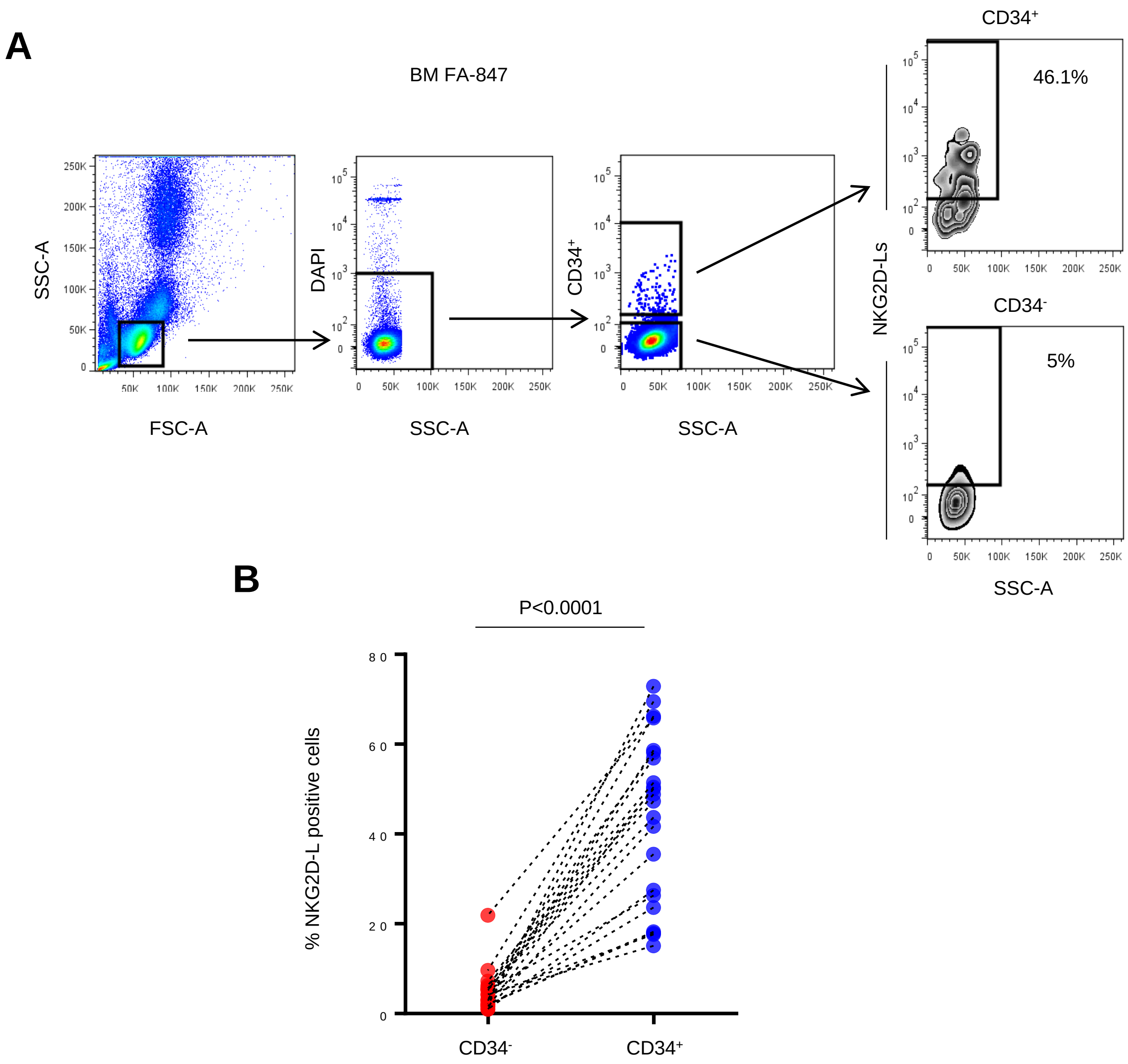

Supplemental Fig. 5

A

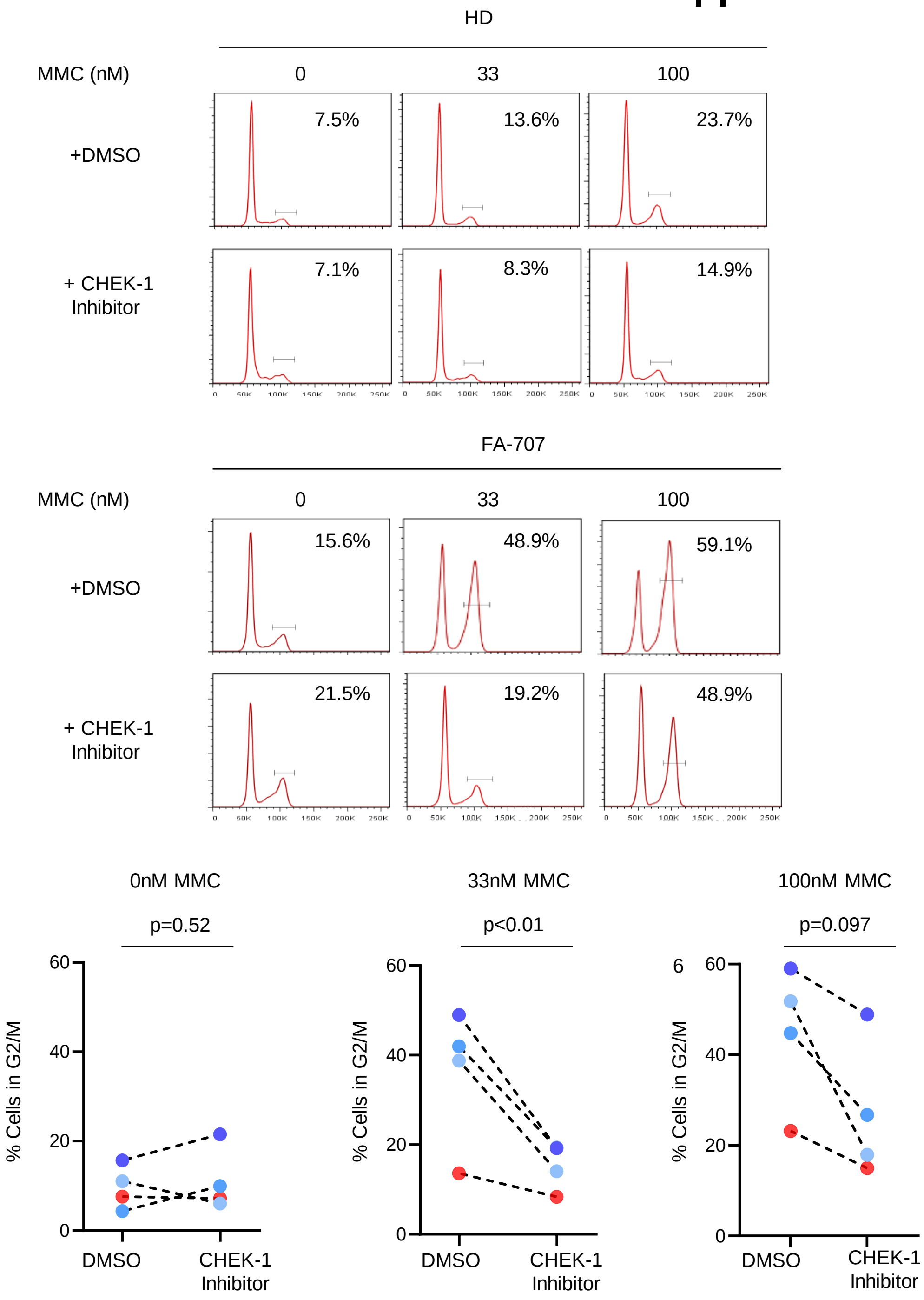

B

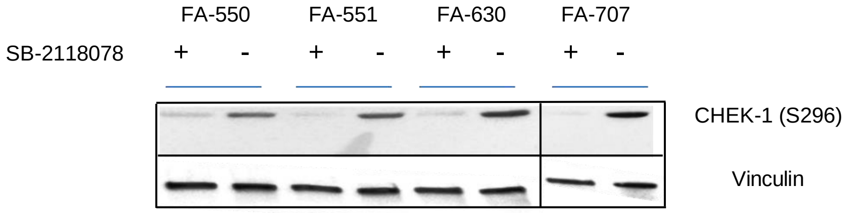

Supplemental Figure 6

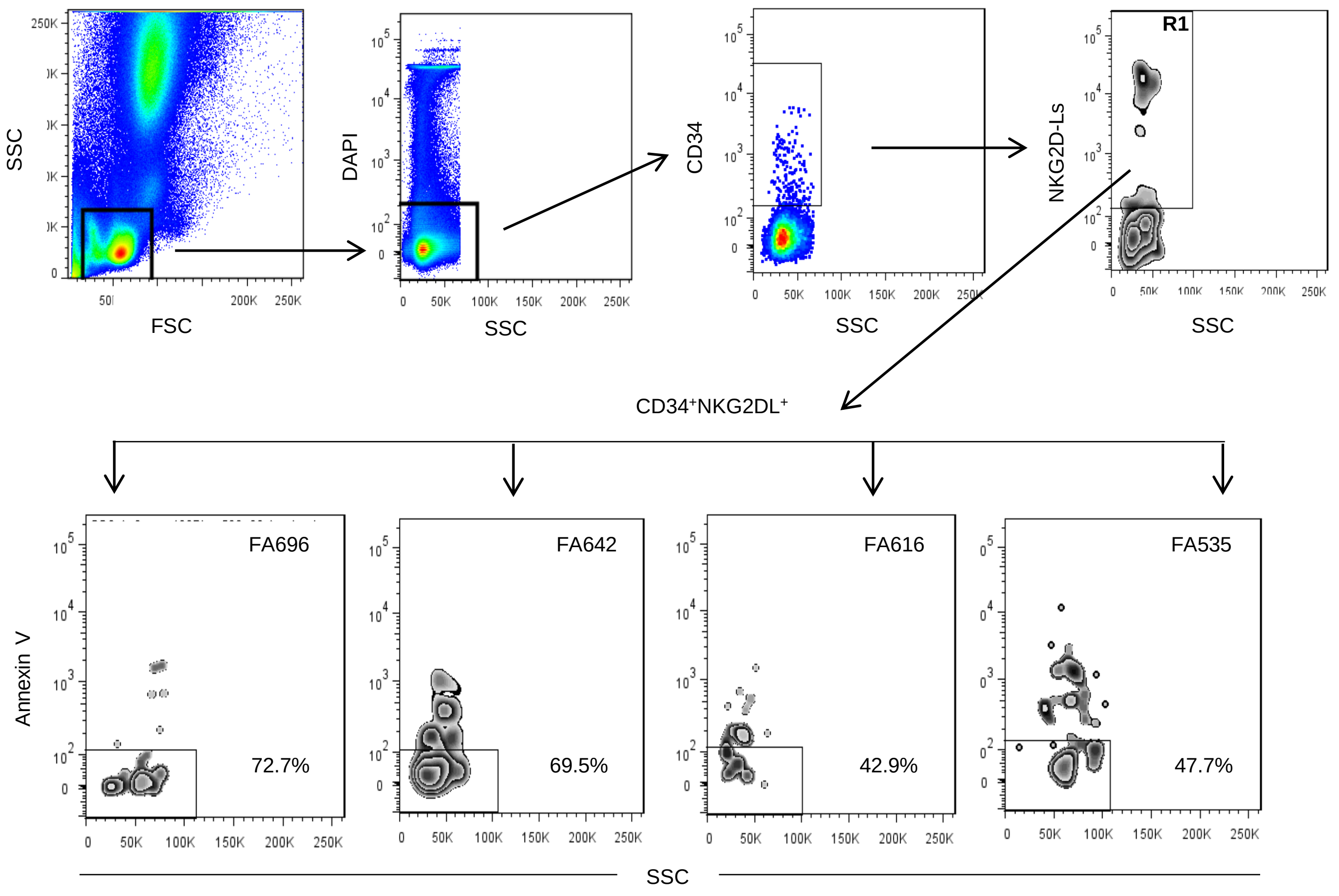

Supplemental Figure 7

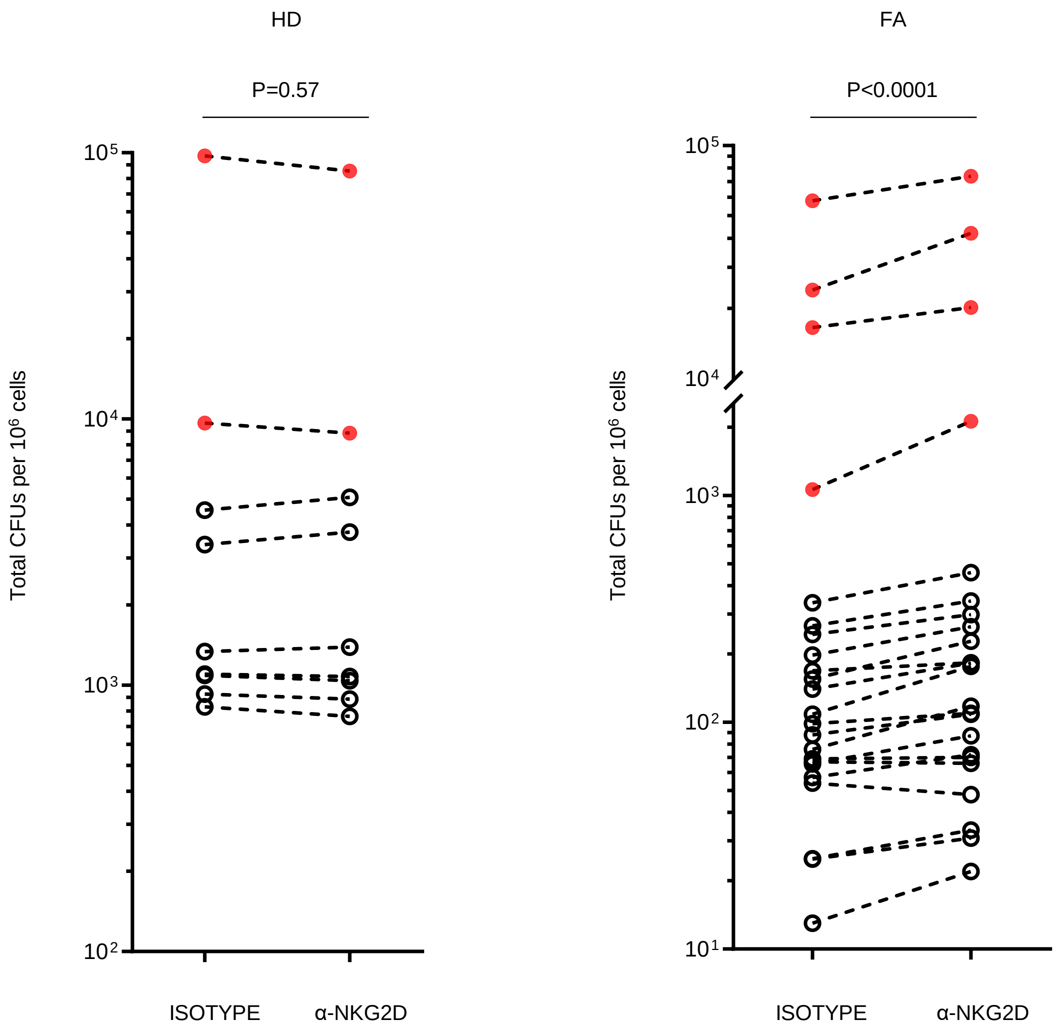

Supplement: Supplemental data [file jci-132-142842-s010.pdf]
